# A multi‐omics approach to overeating and inactivity‐induced muscle atrophy in *db/db* mice

**DOI:** 10.1002/jcsm.13550

**Published:** 2024-07-13

**Authors:** Takuro Okamura, Masahide Hamaguchi, Genki Kobayashi, Takahiro Ichikawa, Yuka Hasegawa, Tomoki Miyoshi, Takafumi Senmaru, Naoko Nakanishi, Ryoichi Sasano, Michiaki Fukui

**Affiliations:** ^1^ Department of Endocrinology and Metabolism Kyoto Prefectural University of Medicine, Graduate School of Medical Science Kyoto Japan; ^2^ Department of Diabetes and Endocrinology Kyoto Okamoto Memorial Hospital Kuze Japan; ^3^ AiSTI Science Co., Ltd. Wakayama Japan

**Keywords:** CUT&Tag, Epigenomics, Genomics, Metabolomics, Muscle atrophy, Obesity

## Abstract

**Background:**

Overeating and inactivity are associated with type 2 diabetes. This study aimed to investigate its pathological basis using integrated omics and *db/db/m*ice, a model representing this condition.

**Methods:**

The study involved housing 8‐week‐old *db/m* and *db/db* mice for 8 weeks. Various analyses were conducted, including gene expression in skeletal muscle and small intestine using next‐generation sequencing; cytokine arrays of serum; assessment of metabolites in skeletal muscle, stool, and serum; and analysis of the gut microbiota. Histone modifications in small intestinal epithelial cells were profiled using CUT&Tag.

**Results:**

Compared with *db/m* mice, *db/db* mice had 22.4% lower grip strength and approximately five times the visceral fat weight (*P* < 0.0001). Serum cytokine arrays showed a 2.8‐fold relative concentration of VEGF‐A in *db/db* mice (*P* < 0.0001) and lower concentrations of several other cytokines. mRNA sequencing revealed downregulation of *Myh* expression in skeletal muscle, upregulation of lipid and glucose transporters, and downregulation of amino acid transporters in the small intestine of *db/db/m*ice. The concentrations of saturated fatty acids in skeletal muscle were significantly higher, and the levels of essential amino acids were lower in *db/db* mice. Analysis of the gut microbiota, 16S rRNA sequencing, revealed lower levels of the phylum Bacteroidetes (59.7% vs. 44.9%) and higher levels of the phylum Firmicutes (20.9% vs. 31.4%) in *db/db* mice (*P* = 0.003). The integrated signal of histone modifications of lipid and glucose transporters was higher, while the integrated signal of histone modifications of amino acid transporters was lower in the *db/db* mice.

**Conclusions:**

The multi‐omics approach provided insights into the epigenomic alterations in the small intestine, suggesting their involvement in the pathogenesis of inactivity‐induced muscle atrophy in obese mice.

## Introduction

Type 2 diabetes is a globally increasing health concern with a complex aetiology influenced by both genetic and environmental factors. Genetic elements contribute to insulin‐related issues, while environmental factors like overeating, inactivity, and obesity amplify the risk of type 2 diabetes. Increased body weight leads to increased insulin resistance, which in turn leads to abnormally elevated blood glucose levels. Sarcopenia is a progressive, systemic skeletal muscle disorder with accelerated loss of muscle mass and function^1^ and has been reported to be a potential cause and consequence of type 2 diabetes.^2^

We have formerly disclosed outcomes from diverse zoological investigations concerning type 2 diabetes and skeletal muscle, demonstrating that *db/db* mice, exhibiting impairments in the leptin receptor and typified by excessive consumption and sedentary behaviour, marked obesity and muscle atrophy.^3^, ^4^ Additionally, *db/db* mice exhibited altered gene expression in the small intestine, affecting fatty acid and amino acid transporters. Dysbiosis and intestinal inflammation resulting from overeating and inactivity were identified as common factors affecting gut microbiota.^5^ The hypothesis proposed suggests that epigenetic changes induced by intestinal inflammation might play a role in the mechanism, leading to diverse traits at an individual level. Epigenomic variation, particularly histone modification, is a regulatory mechanism that alters gene expression while preserving inherited sequences. Acetylation, methylation, and ubiquitination of histone H3 lysine residues are significant in gene expression regulation. However, the relationship between lifestyle‐related diseases like diabetes and epigenetic alterations remains incompletely understood.

Based on the results of various previous studies, we hypothesized that dysbiosis and alterations in intestinal metabolites due to overeating and inactivity alter the epigenome of small intestinal epithelial cells, leading to increased gene expression of long‐chain fatty acid transporters and glucose transporters in the small intestine. As a result, we hypothesized that the absorption of saturated fatty acids and sugar in the small intestine would deviate from a steady state, potentially contributing to the development of lifestyle‐related diseases such as fatty liver and type 2 diabetes. In this study, the following experiments were performed to elucidate the pathological basis of overeating and inactivity‐induced muscle atrophy by performing multi‐omics including genomics, metabolomics, and even epigenomics on samples such as blood, faeces, small intestine, and skeletal muscle.

## Methods

### Murine models

The entirety of experimental methodologies received approval from the Committee for Animal Research at Kyoto Prefectural University of Medicine, Japan (approval designation: M2023‐83). The study, characterized by randomization and investigator blinding, incorporated male *db/m* and *db/db/m*urine models.

**Figure 1:**
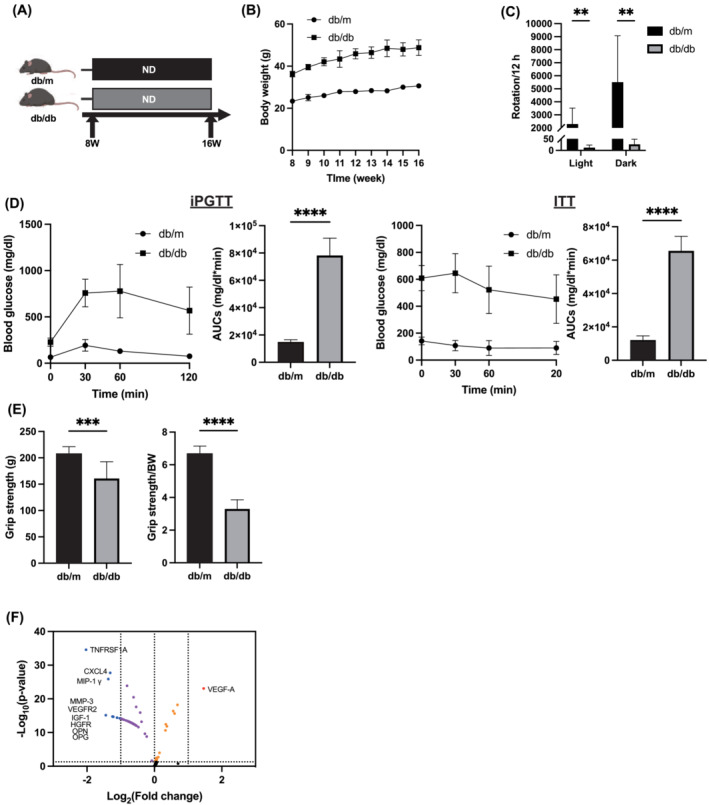
*db/db* mice showed glucose impairment, lower grip strength, and obesity. (A) Mice were sacrificed at 16 weeks of age. (B) Changes in the body weight (*n* = 6). (C) The numbers of rotation in the light and dark phases using the running wheel (*n* = 6). (D) Results of intraperitoneal glucose tolerance test (2 g/kg body weight) for 15‐weeks‐old mice and the area under the curve (AUC) analysis (*n* = 6). Results of insulin tolerance test (0.5 U/kg body weight) for 15‐week‐old mice and the AUC analysis (*n* = 6). (E) Absolute and relative grip strength (*n* = 6). (F) Volcano plot showing the magnitude and significance of differences in serum cytokine concentration levels in *db/m* and *db/db* mice. *Red*, fold change (FC) > 2 and raw *P*‐value < 0.05; *blue*, fc > 0.5 and raw *P*‐value <0.05; *orange*, 1 < fc < 2 and raw *P*‐value <0.05; *purple*, 0.5 < fc < 1 and raw *P*‐value < 0.05. Data are represented as the mean ± SD values. Data were analysed using Welch's *t*‐tests. **P < 0.01, ***P < 0.001, and ****P < 0.0001.

The method for determining sample size is described in Data S1, S2, and S3. Six murine models were allocated to the subsequent groupings: (1) *db/m* models and (2) *db/db/m*odels. Subsequently, the subjects underwent an overnight fasting period and were euthanized at 16 weeks of age via exposure to an anaesthetic amalgamation (comprising 4.0 mg/kg midazolam, 0.3 mg/kg medetomidine, and 5.0 mg/kg butorphanol) (Figure 1A).

### Quantification of voluntary wheel running

Murine subjects were singularly accommodated within enclosures, each equipped with a running wheel. Details are provided in Data S1, S2, and S3.

### Analytical methodologies and tolerance tests for glucose and insulin

Murine models, aged 15 weeks, underwent intraperitoneal glucose tolerance evaluations (iPGTT) (administered at 2 g/kg of body mass) after a fasting period of 16 h and were subjected to insulin tolerance assessments (ITT) (dispensed at 0.5 U/kg of body weight) following a 5‐h fast. Details are provided in Data S1, S2, and S3.

### Assessment of grip strength

The grip strength was quantified utilizing a specialized grip strength meter designated for murine models. Details are provided in Data S1, S2, and S3.

### Serum biochemical analysis

Blood specimens were procured from fasted murine models via cardiac perforation during the process of euthanasia, and the concentrations of alanine aminotransferase (ALT), triglycerides (TG), and total cholesterol were evaluated. Details are provided in Data S1, S2, and S3.

### Histopathological examination of hepatic and epididymal white adipose tissues

Histopathological examination was performed by collecting liver and epididymal white adipose tissues (tissues to evaluate fatty liver and adipocyte size. Details are provided in Data S1, S2, and S3.

### Cytokine antibody arrays

The relative expression levels of 96 cytokines within mouse serum were meticulously assessed. Details are provided in Data S1, S2, and S3.

### Histopathological examination of soleus muscular tissue

For assessment of the skeletal muscle area, tissue from the soleus muscle was procured from euthanized murine models. Details are provided in Data S1, S2, and S3.

### Analysis of mRNA sequencing of the plantaris muscular tissue and jejunum

Details are provided in Data S1, S2, and S3. To scrutinize the functional enrichment of prolific genes within cells and nuclei, each gene was ranked based on log fold change, and gene set enrichment analyses (GSEA) were conducted on gene markers derived from previous studies.^6^ Within these analyses, a positive normalized enrichment score (NES) signifies gene enrichment, whereas a negative NES denotes gene depletion.

### Protein extraction and Western blot analysis

Protein was extracted from the gastrocnemius muscle and small intestine, and Western blot analysis was performed. Details are provided in Data S1, S2, and S3.

### Histopathological evaluation of jejunum and colon

Jejunum and colon samples were collected and stained for histological evaluation of the small and large intestines. Details are provided in Data S1, S2, and S3.

### Isolation of mononuclear cells from murine small intestine and flow cytometric analysis

Mononuclear cells were extracted from the mucosa‐specific layer of the small intestine and flow cytometric analysis was performed. Details are provided in Data S1, S2, and S3. Innate immunity involves various cells, including innate lymphoid cells (ILCs), a subset of lymphocytes constituting the T‐cell innate immune system, encompassing ILC1, ILC2, and ILC3. These lymphocytes release cytokines for a prompt response to pathogenic tissue damage and are poised to contribute to subsequent adaptive immunity.^7^ Disruption of the mucus barrier impacts the quantity of ILC3, a crucial regulator of inflammation and mucosal barrier infections.^8^ The ILC3‐derived IL‐22 plays a pivotal role in maintaining intestinal epithelial integrity.^9^ Conversely, our prior research has indicated an elevation in intestinal ILC1 during inflammation induced by a high‐fat diet.^10^ In addition, ILC3s displayed adaptability, with their functionality being influenced by the presence of the transcription factors RORγt and T‐bet in colitis‐afflicted mice. Upon exposure to cytokines like IL‐12 and IL‐18, there was an increase in ex‐ILC3s, characterized by T‐bet positivity and diminished RORγt positivity, while conventional RORγt‐positive ILC3s decreased. This indicates that ILC3s can dynamically adjust their responses to environmental signals. Previous research has demonstrated that T‐bet‐positive ILC3s, observed in mice with inflammatory bowel disease, produced IFN‐γ and suppressed the production of IL‐17 and IL‐22. Consequently, T‐bet‐positive ILC3s exhibited a function akin to that of ILC1. To assess intestinal inflammation triggered by obesity and inactivity comprehensively, it is essential to examine ILC1, ILC3, and the associated M1/M2 macrophages. The methodologies for flow cytometric analysis of type 1 and 3 innate lymphoid cells and M1/M2 macrophages in the small intestine were conducted following the protocols as previously described^11^, ^12^ (Figure S1A,B). The stained cells were analysed using a Canto II flow cytometer, and the data were analysed using the FlowJo software (version 10; TreeStar, Ashland, OR, USA) (*n* = 6).

### Quantification of metabolites in serum, skeletal muscle, and faeces

Samples, stored at −30°C, including serum (25 μL), faecal matter from the small intestine, and skeletal muscle (20 mg), were utilized for determining metabolite concentrations. A fatty acid methylation kit facilitated methylation analysis, and GC–MS on an Agilent 7890B/7000D system was employed to assess long‐chain fatty acid levels in murine serum, faecal matter, and skeletal muscle tissues. The Varian capillary column (DB‐FATWAX UI; Agilent Technologies) separated fatty acids, with the column temperature profile ranging from 100°C to 240°C. All outcomes were normalized to the C17:0 internal standard.^13^ Gastrocnemius muscle and faeces (20 mg) were homogenized, and the supernatant was processed for extraction. The concentrations of amino acids, organic acids, and SCFAs were determined using GC/MS and normalized to norleucine (0.01 mM) for amino acids and organic acids^14^ and tetradeuteroacetic acid for SCFAs (0.02 mM).^10^ Solid‐phase extraction (SPE) and online SPE‐GC were employed for sample preparation and analysis, respectively. The column temperature profiles were optimized for each set of metabolites. Details are provided in Data S1, S2, and S3.

### Analysis of gut microbiota composition

Appendicular stool samples were collected for gut microbiota analysis and stool DNA was extracted as described in Data S1, S2, and S3.

### Gene ontology and Kyoto Encyclopedia of Genes and Genomes enrichment analysis

Enrichment analysis of Gene Ontology (GO) entries and Kyoto Encyclopedia of Genes and Genomes (KEGG) pathways was performed. The number of genes is the number of genes enriched in GO terms and KEGG pathways. Gene ratio is the percentage of total DEGs in a given GO term or KEGG pathway. Results were sorted according to the proportion of enriched genes in each entry, and the top 10 results were visualized as dot plots. Significance levels were adjusted using the Benjamini–Hochberg method.

### Network analysis and visualization methodology

Co‐abundance gene groups (CAGs) were identified to elucidate the potential groups of bacteria that tend to be abundant or scarce concurrently within the microbial communities. Details are provided in Data S1, S2, and S3.

### Generation of Circos plots

Circos plots were utilized to comprehensively describe the complex relationships and patterns in the multidimensional data set; only pairs with correlation coefficients >0.7 or <−0.7 in CAG, metabolites in stool, and nutrient absorption transporters that showed predominant expression changes between the two groups were drawn.

### Non‐TiE‐UP cleavage under targets and tagmentation

Small intestinal epithelial cells were isolated using previously published protocols.^15^ CUT&Tag was performed using a CUT&Tag‐IT Assay Kit (Active Motif). Zona‐free blastocysts were washed and placed in antibody buffer with primary antibody, digitonin, and protease inhibitor cocktail (PIC) in a 96‐well plate, followed by overnight incubation at 4°C. The cells were transferred to wells with DIG‐Wash buffer, secondary antibody, digitonin, and PIC, then incubated at room temperature. Subsequent steps included incubation in DIG‐300 buffer, pA‐Tn5 transposomes, digitonin, and PIC, followed by tagmentation in individual tubes with tagmentation buffer at 37°C. After tagmentation, ethylenediaminetetraacetic acid (EDTA), sodium dodecyl sulfate (SDS), and proteinase K were added, and DNA purification was performed using SPRIselect beads. Sequencing libraries were PCR amplified, purified, and eluted in DNA purification elution buffer. Paired‐end 38 bp sequence reads (PE38) from Illumina Sequencing were aligned using the BWA algorithm, with alignment information stored in BAM format. MACS3 peak calling identified high‐transposition regions, and fragment density was assessed by dividing the genome into specified bins and determining fragment numbers.^16^ Details are provided in Data S1, S2, and S3.

### Statistical examination

The procured data were subjected to meticulous analysis employing the JMP version 14.0 software (SAS Institute, Cary, NC, USA). The Welch's *t*‐test served as the statistical tool to contrast the dichotomous groups. A *P*‐value inferior to 0.05 was predetermined as the threshold for statistical significance. The illustrative figures were crafted utilizing the GraphPad Prism software (version 9.3.1; San Diego, CA, USA).

## Results

### Comparative physiological and biochemical analyses between *db/db* and *db/m* mice

*db/db* mice weighed significantly more than *db/m* mice (Figure 1B). The quantifiable rotations of the voluntary wheel running by *db/db* mice were significantly lower compared to those by *db/m* mice (Figure 1C). *db/db* mice showed impaired glucose tolerance (Figure 1D), lower grip strength (absolute: 207.0 ± 14.0 g vs. 160.7 ± 31.9 g, relative: 6.7 ± 0.4 vs. 3.3 ± 0.6) (Figure 1E), higher serum lipid levels and liver enzymes (Figure S2A), and higher visceral fat weight (absolute weight: 540 ± 121 mg vs. 2,704 ± 340 mg, relative weight: 0.018 ± 0.004 mg vs. 0.057 ± 0.006 mg) (Figure S2E). The absolute liver weight of *db/db* mice was higher than that of *db/m* mice, but relative liver weights were not significantly different (Figure S2B). In addition, non‐alcoholic fatty liver disease (NAFLD) activity score and Oil Red O staining were also significantly higher in *db/db* mice (Figure S2C,D), and fat cell area was higher in *db/db* mice (Figure S2F). The above results were generally similar to our previous reports.^3^, ^4^ Additionally, variations in serum cytokine concentrations in *db/m* and *db/db* mice were explored utilizing a cytokine antibody array. Serum vascular endothelial growth factor‐A (VEGF‐A) concentrations in *db/db* mice were higher than those in *db/m* mice, while serum concentrations of TNFRSF1A, CXCL4, MIP‐1 γ, MMP‐3, VEGFR2, IGF‐1, HGFR, OPN, and OPG were lower (Figure 1F, Figure S3, and Table S1).

### Morphometric and molecular analysis of skeletal muscle in *db/db* and *db/m* mice

**Figure 2:**
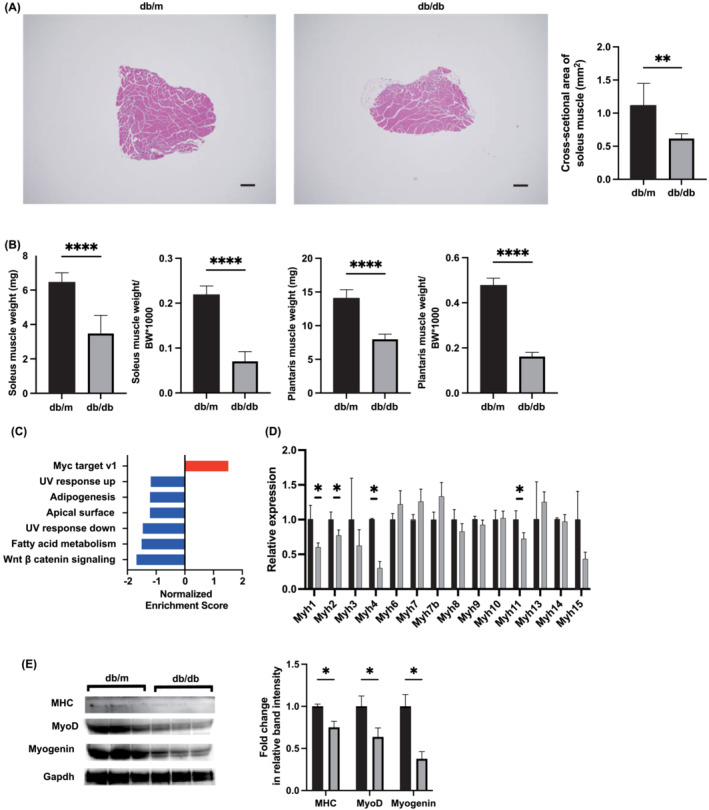
Skeletal muscle loss and changes of gene expression in skeletal muscle in *db/db* mice. (A) Representative images of haematoxylin and eosin‐stained soleus muscle sections. Soleus muscle tissues were collected at 16 weeks of age. The scale bar shows 100 μm. The cross‐sectional area of the soleus muscle (*n* = 6). (B) Absolute and relative soleus muscle weight, absolute and relative plantaris muscle weight in 16‐weeks‐old mice (*n* = 6 in each case). (C) Gene set enrichment analysis (GSEA) in skeletal muscle. The enrichment score for each gene set. ***(D)*** Relative expression of *M*yh (*n* = 6). (E) Western blotting of myosin heavy chain (MHC), MyoD, Myogenin, and Gapdh (*n* = 3). Data are represented as the mean ± SD values. Data were analysed using Welch's *t*‐test. *P < 0.05, **P < 0.01, ***P < 0.001, and ****P < 0.0001.

Depicted in Figure 2A are illustrative instances of haematoxylin and eosin (HE) stained sections of the soleus muscle. The morphometric analysis revealed a lower cross‐sectional area of the soleus muscle in *db/db* mice relative to *db/m* mice (1.12 ± 0.33 mm^2^ vs. 0.62 ± 0.07 mm^2^) (Figure 2A). The absolute and relative weights of the soleus and plantaris muscles in *db/db* mice were notably lower compared to those in *db/m* mice (soleus muscle, 6.4 ± 0.6 mg vs. 3.5 ± 1.1 mg; plantaris muscle, 14.1 ± 1.2 mg vs. 8.0 ± 0.8 mg) (Figure 2B).

Transcriptional data sets were subjected to GSEA to elucidate the underlying biological significance. A selection of significantly enriched gene sets with nominal p values less than 0.05 is depicted, indicating prominence (Figure 2C and Figure S4A). The gene set associated with Myc targets v1 demonstrated augmented expression in *db/db* mice, whereas sets related to Wnt β‐catenin signalling, fatty acid metabolism, UV response down, apical surface, adipogenesis, and UV response up manifested diminished expression.

Furthermore, the expression of a group of genes involved in the myosin heavy chain (*Myh1*, *2*, *3*, *4*, *6*, *7*, *7b*, *8*, *9*, *10*, *11*, *13*, *14*, and *15*) was examined. The expression of *Myh1*, *2*, *4*, and *11* was significantly lower in *db/db* mice than in *db/m* mice (Figure 2D). Protein expression was also examined. The expression of MHC, MyoD, and Myogenin in the skeletal muscle of *db/db* mice was significantly lower than that of *db/m* mice (Figure 2E and Figure S5A).

### Morphological and molecular alterations in intestinal architecture and immune components in *db/db* mice

**Figure 3:**
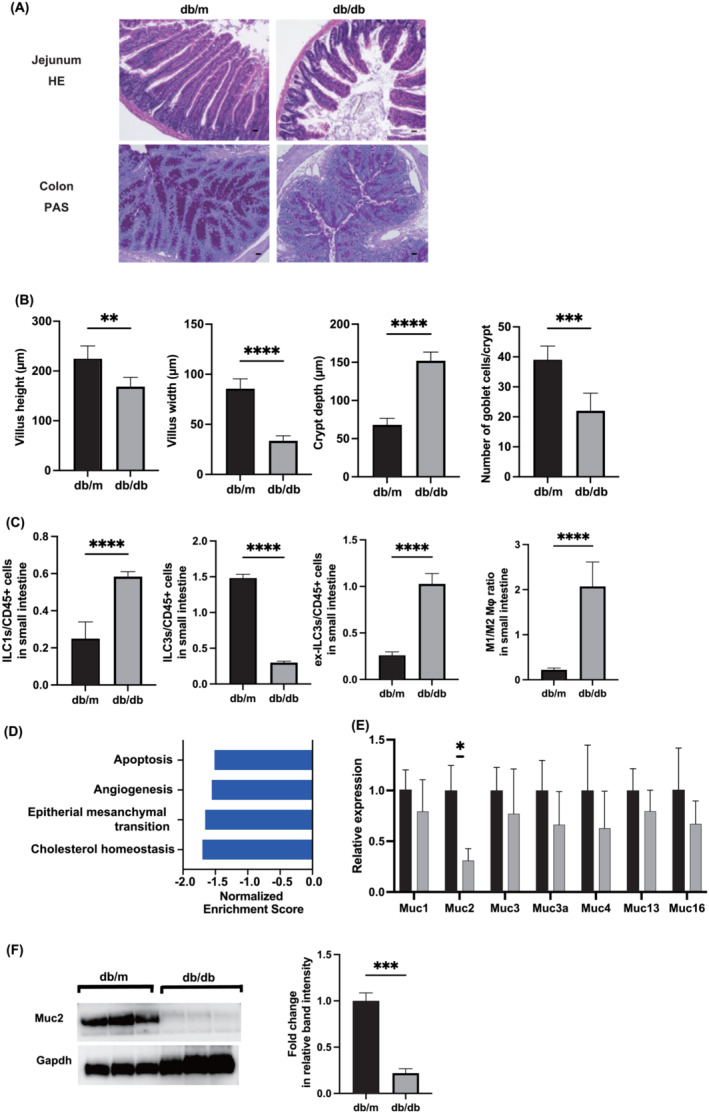
Morphological and genetical changes in small intestinal mucosa in *db/db* mice. (A) Representative images of haematoxylin & eosin (HE)‐stained jejunum and periodic acid Schiff (PAS)‐ stained colon sections. Jejunum and colon tissues were collected at 16 weeks of age. The scale bar shows 100 μm. (B) Villus height and width, and crypt depth (*n* = 6). Total goblet cells/crypt (*n* = 6). (C) Percentages of ILC1s to CD45‐positive cells and ILC3s to CD45‐positive cells, and the ratio of M1 macrophages to M2 macrophages in the small intestine (*n* = 6 in each case). (D) Gene set enrichment analysis (GSEA) in the small intestine. The enrichment score for each gene set. (E) Relative expression of *M*uc (*n* = 6). (F) Western blotting of Muc2 and Gapdh (*n* = 3). Data are represented as the mean ± SD values. Data were analysed using Welch's *t*‐test. *P < 0.05, **P < 0.01, and ***P < 0.001.

Depicted in Figure 3A are exemplary images of jejunum and colon sections subjected to HE and periodic acid‐Schiff (PAS) staining. A conspicuous reduction in both the height and width of the villi, accompanied by an augmentation in crypt depth, was discerned in *db/db* mice (villus height: 224.5 ± 26.0 μm vs. 168.7 ± 18.6 μm; width: 85.5 ± 10.0 μm vs. 33.5 ± 5.1 μm; crypt depth: 68.1 ± 8.4 μm vs. 152.0 ± 11.4 μm) (Figure 3B). A decrement in the count of goblet cells within the crypt, as assessed by PAS staining of the colon, was discerned in *db/db* mice (Figure 3B).

The proportional distribution of ILC1s, ILC3s, and ex‐ILC3s relative to CD45+ cells, in addition to the ratio of M1 macrophages to M2 macrophages, was meticulously assessed within the small intestine. The prevalence of ILC1s and ex‐ILC3s in *db/db* mice significantly was higher than that in *db/m* mice, while the count of ILC3s was lower. The M1/M2 ratio in *db/db* mice was higher than those in *db/m* mice (Figure 3C).

The gene expression datasets underwent GSEA to distill pertinent biological insights. A selection of prominently and significantly enriched gene sets is depicted (Figure 3D and Figure S4B). The gene sets pertinent to cholesterol homeostasis, epithelial‐mesenchymal transition, angiogenesis, and apoptosis manifested downregulation in *db/db* mice. Additionally, the expression of a cohort of genes implicated in mucin production (*Muc1*, *2*, *3*, *3a*, *4*, *13*, and *16*) was scrutinized; notably, the expression of Muc2 was significantly attenuated in *db/db* mice compared to *db/m* mice (Figure 3E). A congruent pattern was observed in the analysis of protein expression (Figure 3F and Figure S5B).

### Analysis of metabolites in the serum, skeletal muscle, and faeces

**Figure 4:**
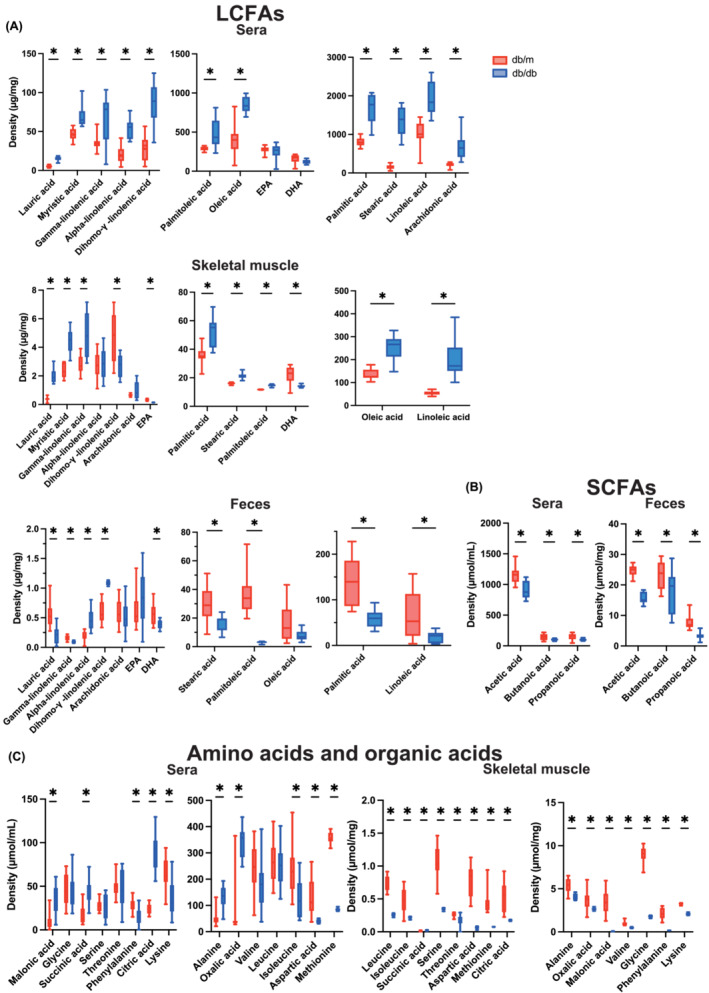
Metabolites in sera, skeletal muscle, and faeces. (A) Concentrations in long‐chain fatty acids (LCFAs) in sera, skeletal muscle, and faeces. (B) Concentrations in short‐chain fatty acids (SCFAs) in sera and faeces. (C) Concentrations in amino acids and organic acids in sera and skeletal muscle (*n* = 6). Data are represented as the mean ± SD values. Data were analysed using Welch's *t*‐test. *P < 0.05.

The levels of long‐chain fatty acids in serum, skeletal muscle, and faeces were scrutinized utilizing a GC/MS system. The *db/db* mice exhibited higher levels of saturated fatty acids such as lauric acid, myristic acid, palmitic acid, and stearic acid in serum and skeletal muscle compared to *db/m* mice. Conversely, the concentrations of saturated fatty acids in the faeces of *db/db* mice were lower relative to those of *db/m* mice. Monounsaturated fatty acids like palmitoleic acid and oleic acid, along with saturated fatty acids, were higher in serum and skeletal muscle in *db/db* mice, yet were lower in faeces. Although the pattern was irregular for polyunsaturated fatty acids, the concentrations of EPA and DHA were attenuated in the skeletal muscle of *db/db* mice relative to *db/m* mice (Figure 4A and Table S2). The levels of acetic acid, butanoic acid, and propanoic acid in the serum and faeces of *db/db* mice were lower than those of *db/m* mice (Figure 4B and Table S5). In serum, the levels of alanine, oxalic acid, malonic acid, succinic acid, serine, and citric acid in *db/db* mice were higher than those in *db/m* mice. In contrast, the levels of valine, leucine, isoleucine, glycine, threonine, aspartic acid, methionine, phenylalanine, and lysine in *db/db* mice were lower than those in *db/m* mice. In skeletal muscle, barring succinic acid, the concentrations of amino acids and organic acids in *db/db* mice were higher than those in *db/m* mice (Figure 4C and Table S3).

### Composition of microbiota in *db/m* and *db/db* mice

**Figure 5:**
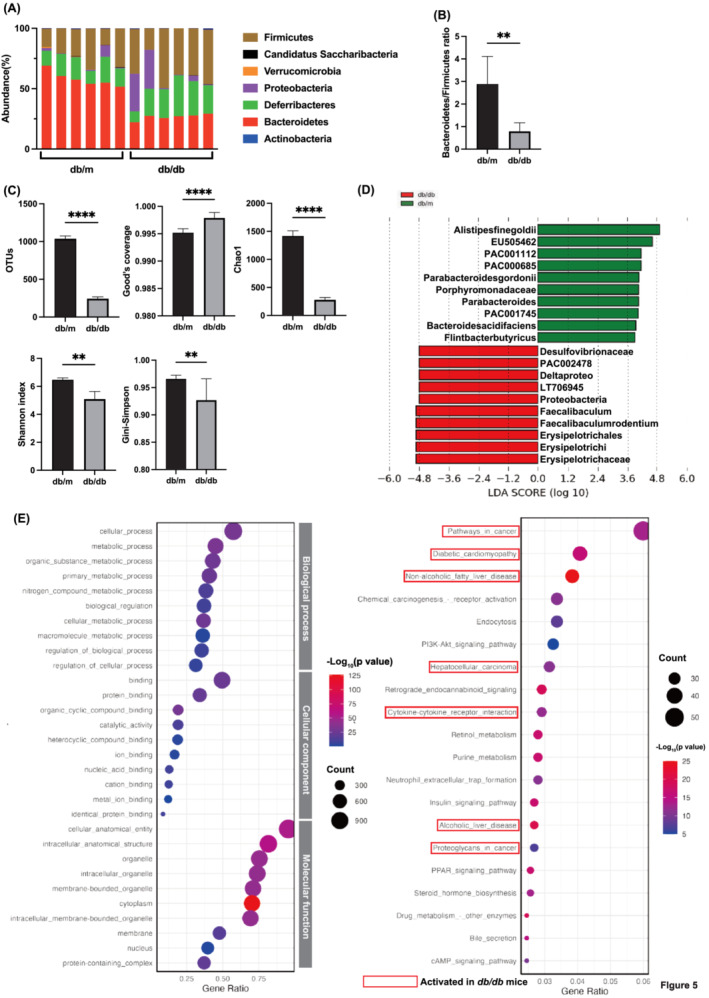
Components of the gut microbiota. (A) Relative abundance of gut microbiota at the phylum levels (*n* = 6). (B) Bacteroidetes/Firmicutes ratio (*n* = 6). (C) OTUs, Good's coverage, Chao1, Shannon‐index, and Gini‐Simpson index, and (*n* = 6). (D) LEfSe identified the taxa with the greatest differences in abundance between *db/m* and *db/db* mice. At the genus level, taxa enriched in *db/db* mice are indicated by a negative LDA score (red), and *db/m* mice enriched taxa are indicated by a positive score (green). (E) Dot plots of GO enrichment analysis including the top 10 significant enrichment terms of three domains (ranked by gene ratio): Biological process, cellular component, and molecular function. (F) Dot plot of KEGG pathways (ranked by gene ratio): Biological process, cellular component, and molecular function. The dot size represents the number of genes belonging to each pathway. The colour gradient is related to the level of significance, adjusted with the Benjamini–Hochberg method. The x‐axis represents the gene proportion enriched in each entry, and the y‐axis shows the enrichment degree according to the adjusted *P*‐value. Data are the mean ± SD values. Data were analysed using Welch's *t*‐test. *P < 0.05, **P < 0.01, ***P < 0.001, and ****P < 0.0001.

An analysis of phyla abundance between the two groups revealed an elevated presence of the Bacteroidetes phylum in *db/m* mice compared to *db/db* mice, accompanied by a reduced abundance of the Firmicutes phylum (Figure 5A,B). The count of operational taxonomic units (OTUs) in *db/db* mice was lower than in *db/m* mice (Figure 5C), with Good's coverage exceeding 97% in both groups. Metrics assessing diversity, including Chao1, Shannon index, and Simpson index, indicated a decrease in *db/db* mice compared to *db/m* mice (Figure 5C).

The LEfSe algorithm was employed to identify the specific taxa that were variably distributed between *db/m* and *db/db* mice. Sixty‐seven taxa were over‐represented (including the family *Erysipelotrichaceae*, *Faecalibaculumrodentium* sp., nov., *Alistipesinops* sp. nov., and Romboutsiatimonensis sp. nov.) and 141 taxa were under‐represented (including *Alistipes finegoldii* sp., nov., *Parabacteroidesgordonii* sp., nov., and *Bacteroidesacidifaciens* sp., nov), and in *db/db* mice (Figure 5D and Figure S6).

To elucidate the function and enrichment pathways of potential genes in *db/m* and *db/db* mice, GO term and KEGG pathway enrichment analysis was performed. In GO enrichment analyses, the top 10 terms of biological process, cellular component, and molecular function were visualized in dot plots, and in KEGG enrichment analysis, the top 20 pathways were visualized in dot plots, respectively (Figure 5E,F). The redder the colour of the dots, the lower the *P*‐value, indicating a higher enrichment of GO terms and KEGG pathways. The results indicated that the cellular process, metabolic process, and organic substance metabolic process are closely related to the biological process of sarcopenia obesity. Furthermore, more significant enrichment was found in protein binding and organic cyclic compound binding for terms of cellular component and in the cellular anatomical entity, intracellular anatomical, and organelle for terms of cellular components (Figure 5E). KEGG analysis showed 209 statistically significant relative pathways, and the top 20 significant enriched potential pathways with the highest gene ratio were shown in a dot plot diagram including ‘pathways in cancer’, ‘diabetic cardiomyopathy’, ‘NAFLD’, and ‘cytokine‐cytokine receptor interaction’ (Figure 5F). These results indicated that overeating and inactivity were involved in the development of sarcopenia obesity via multiple targets and multiple pathways involved in malignancy, metabolic disorders, and inflammation.

### Differential gene expression and correlation of nutrient transporters and microbial clusters

**Figure 6:**
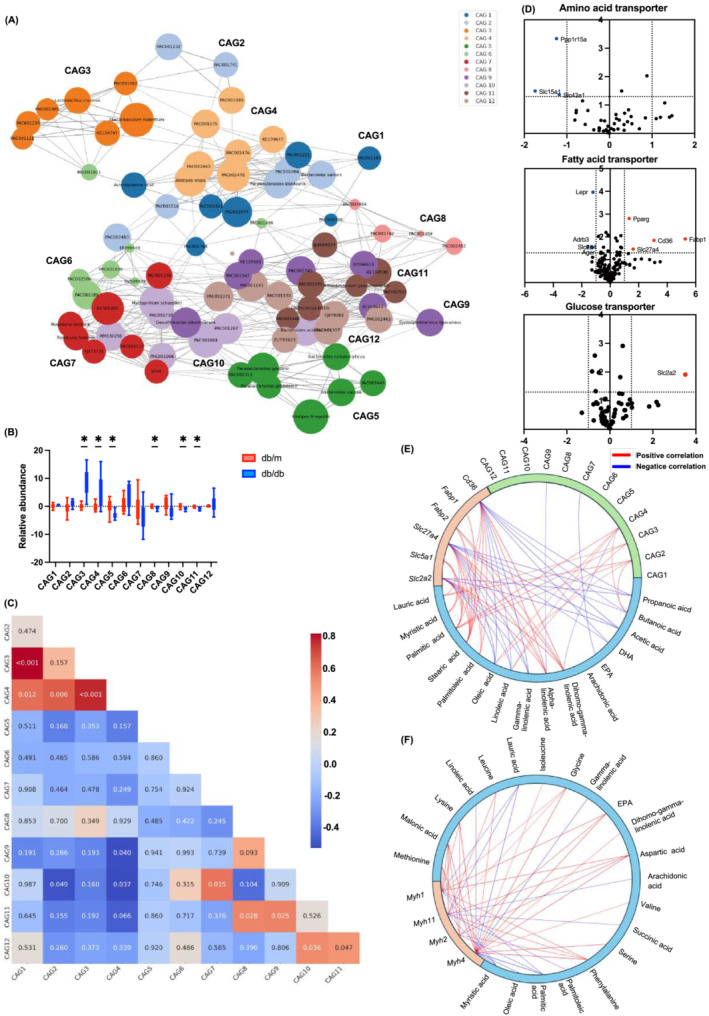
Correlation analyses between gut microbiota, metabolites, and genes. (A) Network diagram at the metagenomic operational taxonomic unit (mOTU) level. This is illustrating the abundance of mOTUs in different groups, based on significantly changed CAGs. The size of the node represents the average abundance of each mOTU. Lines between nodes represent correlations, with the width of the line indicating the magnitude of the correlation and purple representing a positive correlation. Different colours have been used to distinguish CAGs. (B) Relative abundances (Log_2_) of 12 CAGs. *Blue*, *db/m*; *red*, *db/db*. (C) Pearson's correlation coefficient analysis between CAGs with *P*‐value. (D) Volcano plot showing the magnitude and significance of differences in gene expression levels related to amino acid transporter, fatty acid transporter, and glucose transporter of the small intestine in *db/m* and *db/db* mice. *Red*, fold change (fc) > 2 and raw *P*‐value <0.05; *blue*, fc > 0.5 and raw *P*‐value <0.05. (E) Circos plot displaying co‐abundance gene groups (CAGs), metabolites, and genes in the small intestine. (F) Circos plot displaying metabolites and genes. Red links stand for positive correlations and blue links stand for negative correlations. Data are the mean ± SD values. Data were analysed using Welch's *t*‐test. *P < 0.05, **P < 0.01, ***P < 0.001, and ****P < 0.0001.

The mOTUs were then clustered into 12 CAGs to create a coexisting network (Figure 6A and Figure S7). The top 7 were selected for inclusion in the network visualization. CAG 3 and 4 were notably more prevalent in the *db/db* mice than the *db/m* mice, while CAG 5, 8, 10, and 11 were less prevalent (Figure 6B). *Lactobacillus murinus* and *Faecalibaculum rodentium* in CAG3 and *Alistipes inops* in CAG4 were reported to be increased in the intestines of mice fed a high‐fat diet.^17^, ^18^ Positive correlations were observed among CAGs 1, 2, 3, and 4, and among CAGs 7, 8, 9, 10, 11, and 12. These two CAG groups were negatively associated with each other (Figure 6C).

Volcano plots depicted gene expression of nutrient transporters in the small intestine for amino acids, fatty acids, and carbohydrates. Compared with *db/m* mice, *Ppp1r15a*, *Slc15a1*, and *Slc43a1* were downregulated in amino acid transporters, while *Fabp1*, *Pparg*, *Cd36*, *Slc27a4*, and *Slc2a2* were upregulated in fatty acid transporters, and *Slc2a2* was upregulated in carbohydrate transporters (Figure 6D).

A Circos plot (Figure 6E) showed correlations between *Fabp1*, *Pparg*, *Cd36*, *Slc27a4*, *Slc15a1*, *Slc2a2* genes, metabolites, and CAGs. CAG3 correlated positively with lauric acid and arachidonic acid, while CAG4 correlated positively with palmitic acid, lauric acid, linolenic acid, and oleic acid. *Fabp1*, *Cd36*, *Slc27a4*, and *Slc2a2* displayed various positive associations with fatty acids and negative associations with acetic acid. Conversely, *Slc15a1* showed positive associations with DHA and propanoic acid and negative associations with myristic acid.

Finally, the correlation between the expression of myosin heavy chain‐related genes and metabolites in skeletal muscle was plotted in a circos plot (Figure 6F). Saturated fatty acids, such as lauric acid, myristic acid, and palmitic acid, were negatively associated with *Myh1*, *Myh2*, and *Myh4*, whereas EPA and amino acids, such as valine, leucine, isoleucine, glycine, methionine, phenylalanine, and lysine, were positively associated.

### Comparative analysis of H3K9ac distribution and peaks in genomic regions

**Figure 7:**
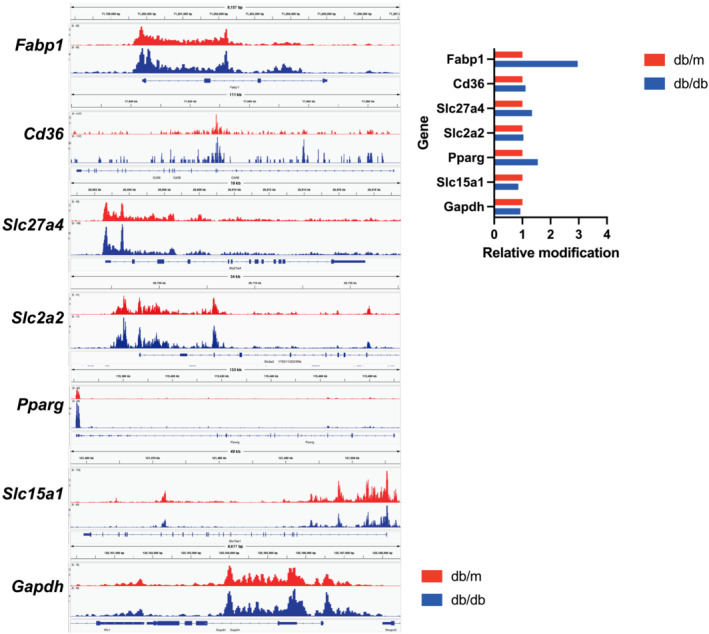
Global histone modifications via gene bodies in small intestinal epithelial cells. (A) Genome browser screenshot showing *db/m* (red) and *db/db* (blue) CUT&Tag signal from experiments in small intestinal epithelial cells; *Fabp1*, *Cd36*, *Slc27a4*, *Slac2a2*, *Pparg*, *Slc15a1*, and *Gapdh*. (B) Integral value of the signal peak. *db/db* mouse value was set to 1.

The pie chart (Figure S8A) displays histone labelling peaks for H3K9ac in different genome regions, with introns having the highest peaks. Scatterplots (Figure S8B) compare tag numbers between *db/db* and *db/m* mice, with a slope of 1.1802. Furthermore, the slope, an index of the average ratio of the number of tags between the two samples, was 1.1802. Tag distribution across target regions for Gene bodies (with 2 kb flanking regions), merged peak regions (= all peak regions; ±5 kb), and transcription start sites (TSS; ±5 kb), both mean plots and heat maps were displayed. By default, heat maps clustered the data using the k‐means algorithm and sorted by decreasing mean within each cluster (Figure S8C). Genebodies, merged peak regions, and promoters in *db/db* mice were higher than those in *db/m* mice. Three different charts were generated to compare peak sizes between the two sample groups. The metric used for these charts was the number of merged peak region tags; the VENN diagram showed the number of merged regions containing peaks from samples in each overlap category (Figure S8D,E). To quantitatively compare the levels of epigenetic modifications in specific genomic regions, we calculated the integrated signal of histone modifications across these regions. The integrated signal of histone modifications of *Fabp1*, *Cd36*, *Slc27a4*, and *Pparg*, which were highly expressed in the *db/db* group by mRNA‐seq, was high in the *db/db* group, while the integrated signal of histone modifications of *Slc15a1*, which was low in the *db/db* group by mRNA‐seq, was low in the *db/db* group (Figure 7).

## Discussion

We observed a notable decline in muscle strength and mass in *db/db* mice, known for severe obesity and glucose intolerance due to overeating and inactivity. Additionally, an increase in inflammatory cells in the small intestine's mucosal lining and alterations in the intestinal microbiota were noted. Multi‐omics analysis indicated a correlation between intestinal metabolites and genes related to nutrient absorption. Lastly, histone acetylation of fatty acid absorption‐related genes was significantly enhanced in *db/db* mice.

The *db/db* mice developed prominent obesity and impaired glucose tolerance, as previously reported, as well as fatty liver and increased visceral fat.^19^, ^20^ Serum cytokine array results revealed elevated VEGF‐A expression, known for inducing angiogenesis and promoting vascular endothelial cell functions. This elevation suggested systemic atherosclerosis linked to impaired glucose tolerance.^S1–3^ On the other hand, serum expression of TNFRSF1A, CXCL4, MIP‐1 γ, MMP‐3, VEGFR2, IGF‐1, HGFR, OPN, and OPG was significantly lower in *db/db* mice. Among them, MIP‐1γ, MMP‐3, VEGFR2, IGF‐1, and HGFR are associated with obesity and OPN and OPG are associated with atherosclerotic diseases. However, the roles of individual genes and receptors are complex, and multiple factors influence the development and progression of obesity and related diseases. The role of those cytokines and chemokines is only one of them, and their interactions with other genes and environmental factors must also be considered. Therefore, although several studies have suggested an association with obesity, the detailed mechanisms and clinical significance need further study.

The concentration of saturated fatty acids increased significantly in *db/db* mouse skeletal muscle, along with altered gene and protein expression. GSEA revealed reduced expression of fatty acid metabolism‐related genes. Additionally, *Myh1*, *2*, *4*, and *11* isoforms of myosin heavy chains showed decreased expression in *db/db* mice. mRNA sequencing of plantaris muscle, a fast‐twitch muscle, demonstrated high expression of *Myh1*, *2*, and *4*, known for their involvement in skeletal muscle development.^21^ Correlation analysis between metabolites and genes indicated a negative association between saturated fatty acids and Myh1, 2, and 4 expressions, suggesting their role in obesity related inactivity‐induced muscle atrophy.

In the lamina propria of the small intestine, the number of ILC3, which secretes IL‐22 and promotes mucin secretion from goblet cells, was decreased in the intrinsic layer of the small intestine in *db/db* mice.^22^ In both mRNA‐seq and Western blotting, the expression of Muc2 was significantly reduced in *db/db* mice. Furthermore, inflammatory cells such as ILC1 and M1 macrophages in the lamina propria of the small intestine were increased in *db/db* mice,^23^ and the production of SCFAs was decreased in the faeces of *db/db* mice. Since SCFAs have been reported to promote IL22 secretion from ILC3,^24^ the decrease in SCFAs in the intestine might be one of the mechanisms of intestinal inflammation development in *db/db* mice.

In the analysis of the gut microbiota, *db/db* mice exhibited a significantly lower Bacteroidetes/Firmicutes ratio and reduced diversity, associated with obesity and impaired glucose tolerance.^25^, ^26^ *Lactobacillus murinus* , Faecalibaculum rodentium, and Alistipes inops, identified in CAG3 and CAG4, were elevated in *db/db* mice, correlating positively with a high‐fat diet.^17^, ^18^ Correlation analyses revealed that CAG3 and CAG4, along with *Fabp1*, *Cd36*, *Slc27a4*, and *Slc2a2*, were strongly linked to saturated fatty acids. Conversely, *Slc15a1*, an amino acid transporter, correlated positively with DHA and propionic acid. Enrichment analyses using GO term and KEGG pathways also showed that the gut microbiota associated with cancer, metabolic disorders, and inflammation were involved in the development of overeating and inactivity‐induced muscle atrophy. These findings suggest gut microbiota and intestinal metabolite alterations may influence small intestine gene expression. Gut microbiota impact various metabolic processes, including nutrient absorption, hormone secretion, immune modulation, and metabolite synthesis.^27^ Additionally, the gut microbiome can modulate host gene expression via microbial metabolites, potentially influencing disease onset and progression.^S9^

In this study, we analysed histone acetylation in small intestinal epithelial cells. We observed a lower number of peak regions in *db/db* mice, but mRNA‐seq showed elevated expression of fatty acid and carbohydrate transporters. The integrated signal of histone modifications associated with these transporters was higher in *db/db* mice, while that of amino acid transporters was lower. This suggests that overeating or inactivity may induce epigenomic changes in small intestinal epithelial cells, affecting gene expression. Histone acetylation is generally related to active gene expression, and abnormal histone acetylation/deacetylation is involved in numerous pathological conditions, including inflammatory and degenerative diseases.^28^ While diabetes‐related histone acetylation has been studied in pancreatic islets and intestines,^29^ this is the first study focusing on nutrient absorption transporters in the context of obesity and diabetes onset. The correlation network analysis suggests that saturated fatty acids may positively regulate fatty acid and sugar transporter expression, while polyunsaturated and short‐chain fatty acids may have a negative effect. This epigenomic change is supported by findings demonstrating aggravation of glucose tolerance, obesity, and sarcopenia with increased saturated fatty acids intake in *db/db* mice.^4^

As one limitation, only male mice were used in this experimental system. One reason is that the innate immune system of female mice is reported to be altered by the estradiol cycle.^30^, ^31^ We have shown in our own experiments that estradiol stimulation specifically increases the number of ILC3, and we used male mice to improve the reproducibility of the experiment. Furthermore, it has been reported that macrophages express oestrogen receptors and form a subtype similar to M2 macrophage upon oestrogen stimulation.^32^ Therefore, male mice were used to improve the reproducibility of the experiment.

In summary, multi‐omics analysis and GSEA indicated correlations between intestinal metabolites and the expression of genes related to nutrient absorption in the small intestine. Notably, histone acetylation of fatty acid absorption‐related genes was significantly enhanced in *db/db* mice, suggesting the possibility of epigenomic changes in small intestinal epithelial cells due to overeating or inactivity. The results of this study provide comprehensive insights into the multifaceted changes associated with obesity, glucose intolerance, and muscle atrophy and pave the way for future research and potential therapeutic strategies.

## Conflict of interest

Takuro Okamura, Genki Kobayashi, Takahiro Ichiakawa, Yuka Hasegawa, Tomoki Miyoshi, Ryoichi Sasano, and Naoko Nakanishi declare that they have no conflict of interest. Masahide Hamaguchi has received grants from Asahi Kasei Pharma, Nippon Boehringer Ingelheim Co., Ltd., Mitsubishi Tanabe Pharma Corporation, Daiichi Sankyo Co., Ltd., Sanofi K.K., Takeda Pharmaceutical Company Limited, Astellas Pharma Inc., Kyowa Kirin Co., Ltd., Sumitomo Dainippon Pharma Co., Ltd., Novo Nordisk Pharma Ltd., and Eli Lilly Japan K.K., outside the submitted work. Takafumi Senmaru received personal fees from Eli Lilly Japan K.K., Mitsubishi Tanabe Pharma Co, Daiichi Sankyo Co. Ltd., Kowa Pharma Co., Ltd., Astellas Pharma Inc., Takeda Pharma Co., Ltd., Sanofi K.K., Taisho Toyama Pharma Co., Ltd., Kyowa Kirin Co., Ltd., Kissei Pharma Co., Ltd., MSD K.K., Novo Nordisk Pharma Ltd., Ono Pharma Co., Ltd., AstraZeneca K.K., Mochida Pharma Co. Ltd., TERUMO CORPORATION, Abbott Japan Co. Ltd., outside the submitted work. Michiaki Fukui has received grants from Nippon Boehringer Ingelheim Co., Ltd., Kissei Pharma Co., Ltd., Mitsubishi Tanabe Pharma Co, Daiichi Sankyo Co., Ltd., Sanofi K.K., Takeda Pharma Co., Ltd., Astellas Pharma Inc., MSD K.K., Kyowa Hakko Kirin Co., Ltd., Sumitomo Dainippon Pharma Co., Ltd., Kowa Pharmaceutical Co., Ltd., Novo Nordisk Pharma Ltd., Ono Pharma Co., Ltd., Sanwa Kagaku Kenkyusho Co., Ltd. Eli Lilly Japan K.K., Taisho Pharma Co., Ltd., Terumo Co., Teijin Pharma Ltd., Nippon Chemiphar Co., Ltd., and Johnson & Johnson K.K. Medical Co., Abbott Japan Co., Ltd., and received personal fees from Nippon Boehringer Ingelheim Co., Ltd., Kissei Pharma Co., Ltd., Mitsubishi Tanabe Pharma Corp., Daiichi Sankyo Co., Ltd., Sanofi K.K., Takeda Pharma Co., Ltd., Astellas Pharma Inc., MSD K.K., Kyowa Kirin Co., Ltd., Sumitomo Dainippon Pharma Co., Ltd., Kowa Pharma Co., Ltd., Novo Nordisk Pharma Ltd., Ono Pharma Co., Ltd., Sanwa Kagaku Kenkyusho Co., Ltd., Eli Lilly Japan K.K., Taisho Pharma Co., Ltd., Bayer Yakuhin, Ltd., AstraZeneca K.K., Mochida Pharma Co., Ltd., Abbott Japan Co., Ltd., Medtronic Japan Co., Ltd., Arkley Inc., Teijin Pharma Ltd. and Nipro Cor., outside the submitted work.

## Supporting information


**Figure S1.** Strategy for innate lymphoid cells (ILCs) and M1 and M2 macrophages (A) Representative flow cytometry plots of liver CD45 + Live & Dead‐ Lin‐ CD127 + RORg‐ GATA‐3‐ T‐bet+ ILC1s, CD45 + Live & Dead‐ Lin‐ CD127 + RORg+ GATA‐3‐ ILC3s, and T‐bet+ ILC3s are Ex‐ILC3s in each group at 16‐weeks of age. (B) CD45 + F480 + CD206 + CD11c‐ M1 macrophages and CD45 + F480 + CD206‐ CD11c + M2 macrophages.
**Figure S2.** Liver and epididymal fat histological analyses.
**Figure S3.** Cytokine array.
**Figure S4.** GSEA analysis in skeletal muscle and small intestine.
**Figure S5.** Full gels for Western blot images of skeletal muscle and small intestine for quantification in Figure 2E and 3F.
**Figure S6.** LADA scroes.
**Figure S7.** Network diagram at the Metagenomic Operational Taxonomic Unit (mOTU) level. This is illustrating the abundance of mOTUs in different groups, based on significantly changed CAGs. The size of the node represents the average abundance of each mOTU. Lines between nodes represent correlations, with the width of the line indicating the magnitude of the correlation and purple representing a positive correlation. Different colours have been used to distinguish CAGs.
**Figure S8.** Global histone modifications via gene bodies in small intestinal epithelial cells.

**Table S1.** Serum cytokine array.

**Table S2.** Concentration of long chain fatty acids in serum, skeletal muscle, and faeces.

**Table S3.** Concentration of short chain fatty acids in serum and faeces and concentration of amino acids and organic acids in serum and skeletal muscle.

**Data S1.** Supporting Information

**Data S2.** Supporting Information

**Data S3.** Supporting Information
