# Supplementary material for: A multi‐omics approach to overeating and inactivity‐induced muscle atrophy in db/db mice
Source: J Cachexia Sarcopenia Muscle. 2024 Jul 13;15(5):2030–45. doi: 10.1002/jcsm.13550 (PMC11446703; doi:10.1002/jcsm.13550)
Supplement: Supplementary file 1 — Figure S1. Strategy for innate lymphoid cells (ILCs) and M1 and M2 macrophages (A) Representative flow cytometry plots of liver CD45 + Live & Dead‐ Lin‐ CD127 + RORg‐ GATA‐3‐ T‐bet+ ILC1s, CD45 + Live & Dead‐ Lin‐ CD127 + RORg+ GATA‐3‐ ILC3s, and T‐bet+ ILC3s are Ex‐ILC3s in each group at 16‐weeks of age. (B) CD45 + F480 + CD206 + CD11c‐ M1 macrophages and CD45 + F480 + CD206‐ CD11c + M2 macrophages. Figure S2. Liver and epididymal fat histological analyses. Figure S3. Cytokine array. Figure S4. GSEA analysis in skeletal muscle and small intestine. Figure S5. Full gels for Western blot images of skeletal muscle and small intestine for quantification in Figure 2E and 3F. Figure S6. LADA scroes. Figure S7. Network diagram at the Metagenomic Operational Taxonomic Unit (mOTU) level. This is illustrating the abundance of mOTUs in different groups, based on significantly changed CAGs. The size of the node represents the average abundance of each mOTU. Lines between nodes represent correlations, with the width of the line indicating the magnitude of the correlation and purple representing a positive correlation. Different colours have been used to distinguish CAGs. Figure S8. Global histone modifications via gene bodies in small intestinal epithelial cells. [file JCSM-15-2030-s004.pdf]

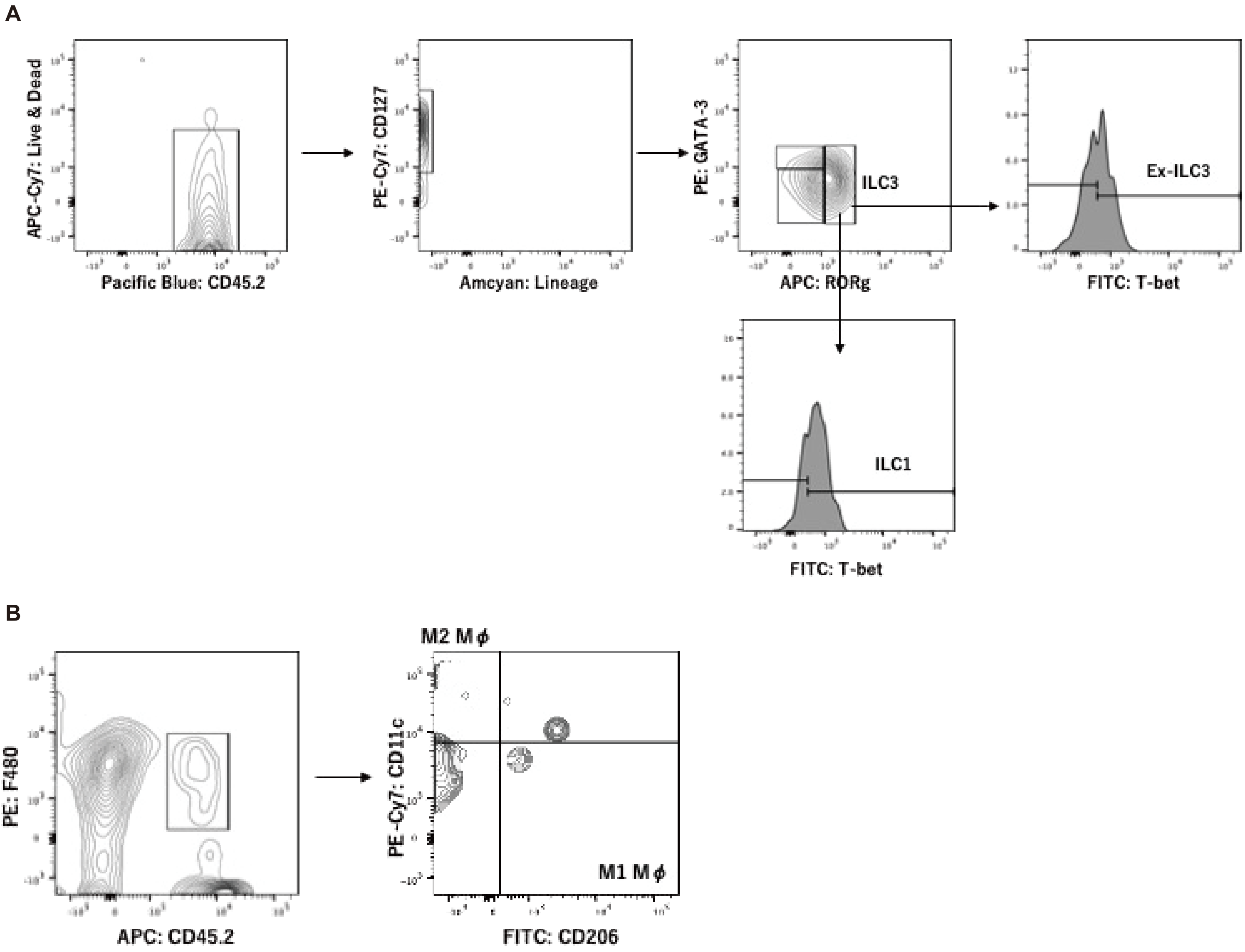

Supplementary Figure 1. Strategy for innate lymphoid cells (ILCs) and M1 and M2 macrophages

(A) Representative flow cytometry plots of liver CD45+ Live & Dead- Lin- CD127+ RORg- GATA-3- T-bet+ ILC1s, CD45+ Live & Dead- Lin- CD127+ RORg+ GATA-3- ILC3s, and T-bet+ ILC3s are Ex-ILC3s in each group at 16-weeks of age. (B) CD45+ F480+ CD206+ CD11c- M1 macrophages and CD45+ F480+ CD206- CD11c+ M2 macrophages.

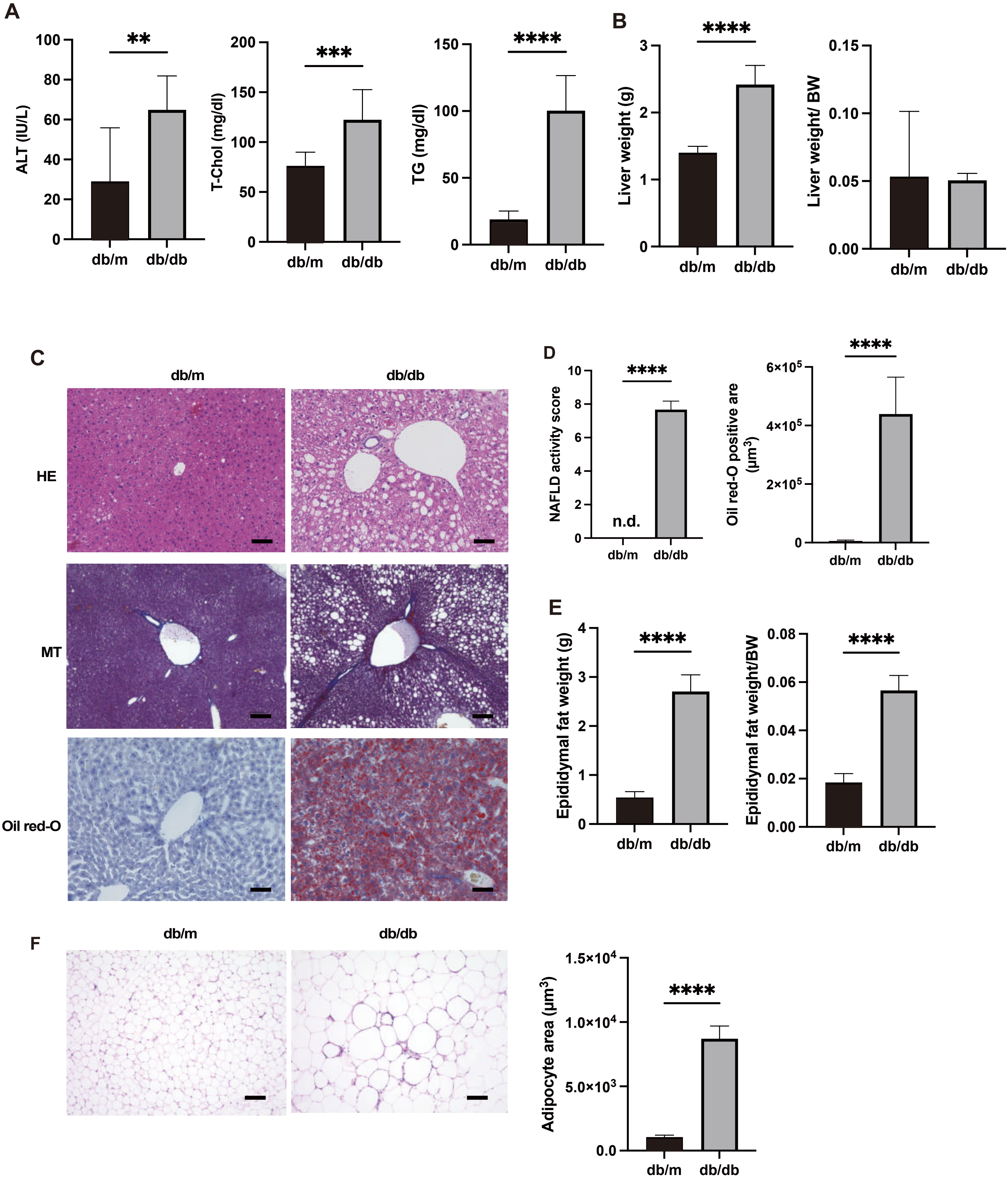

Supplementary Figure 2. Liver and epididymal fat histological analyses

(A) Serum alanine aminotransferase (ALT), total cholesterol (T-Chol), and triglyceride (TG) levels (n = 6).

(B) Absolute and relative liver weight (n = 6).

(C) Representative images of hematoxylin & eosin-stained, Masson-Trichrome-stained, and Oil red-O stained liver sections. Liver tissues were collected at 16 weeks of age. The scale bar shows 100 μm.

(D) NAFLD activity score (n = 6). Oil red-O positive area (μm^3) (n = 6).

(E) Absolute and relative epididymal fat weight (n = 6).

(F) Representative images of hematoxylin & eosin-stained epididymal fat sections. Adipocyte area (μm^3) (n = 6)

Data are represented as the mean ± SD values. Data were analyzed using paired t-test. ****$p < 0.0001$.

C3_db/m

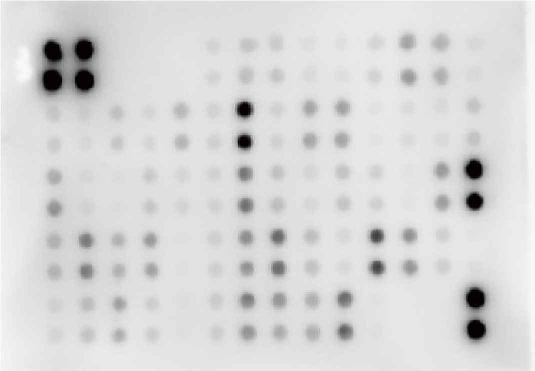

C3_db/db

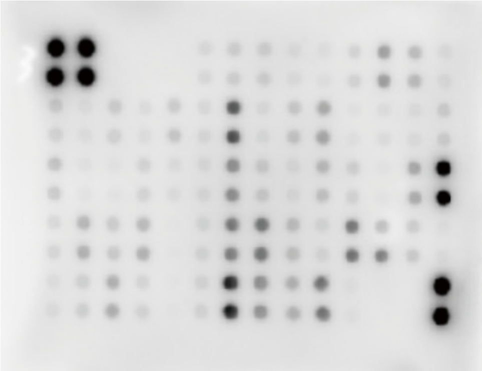

Each antibody is spotted in duplicate vertically

	A	B	C	D	E	F	G	H	I	J	K	L	M	N
1<br>2	POS	POS	NEG	NEG	BLANK	Axl	BLC (CXCL13)	CD30 Ligand (TNFSF8)	CD30 (TNFRSF8)	CD40 (TNFRSF5)	CRG--2	CTACK (CCL27)	CXCL16	Eotaxin-1 (CCL11)
3<br>4	Eotaxin-2 (CCL24)	Fas Ligand (TNFSF6)	Fractalkine (CX2CL1)	GCSF	GM-CSF	IFN-gamma	IGFBP-3	IGFBP-5	IGFBP-6	IL-1 alpha (IL-1 F1)	IL-1 beta (IL-1 F2)	IL-2	IL-3	IL-3 R beta
5<br>6	IL-4	IL-5	IL-6	IL-9	IL-10	IL-12 p40/p70	IL-12 p70	IL-13	IL-17A	KC (CXCL1)	Leptin R	Leptin	LIX	L-Selectin (CD62L)
7<br>8	Ltn (XCL1)	MCP-1 (CCL2)	MCP-5	M-CSF	MIG (CXCL9)	MIP-1 alpha (CCL3)	MIP-1 gamma	MIP-2	MIP-3 beta (CCL19)	MIP-3 alpha (CCL20)	PF-4 (CXCL4)	P-Selectin	RANTES (CCL5)	SCF
9<br>10	SDF-1 alpha	TARC (CCL17)	I-309 (TCA-3/CCL1)	TECK (CCL25)	TIMP-1	TNF alpha	TNF RI (TNFRSF1A)	TNF RII (TNFRSF1B)	TPO	VCAM-1 (CD106)	VEGF-A	BLANK	BLANK	POS

C4_db/m

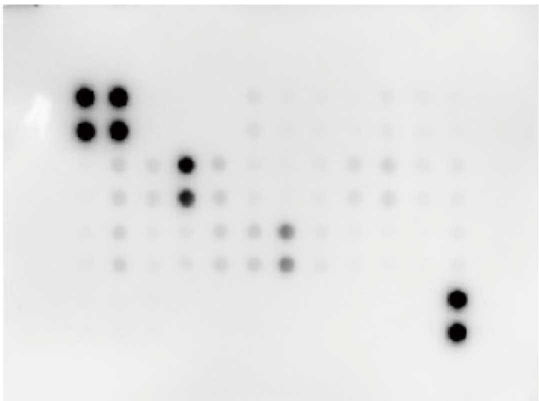

C4_db/db

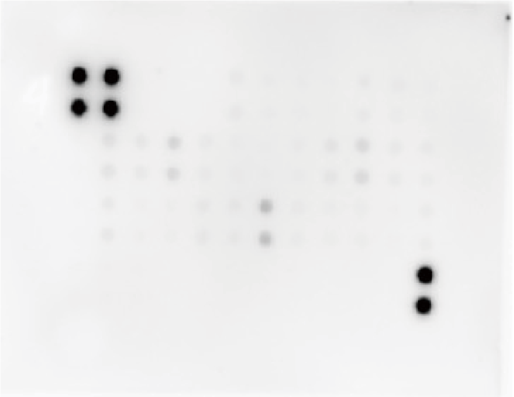

Each antibody is spotted in duplicate vertically

	A	B	C	D	E	F	G	H	I	J	K	L
1<br>2	POS	POS	NEG	NEG	BLANK	bFGF	CD26 (DPPIV)	Dtk	E-Selectin	Fc gamma RIIB	Flt-3 Ligand	GITR (TNFRSF18)
3<br>4	HGFR	ICAM-1 (CD54)	IGFBP-2	IGF-1	IGF-2	IL-15	IL-17 RB	IL-7	I-TAC (CXCL11)	Lungkine (CXCL15)	MDC (CCL22)	MMP-2
5<br>6	MMP-3	OPN (SPP1)	OPG (TNFRSF11B)	Pro-MMP-9	Resistin	Shh-N	TCK-1 (CXCL7)	TIMP-2	TRANCE (TNFSF11)	TROY (TNFRSF19)	TSLP	VEGFR1
7<br>8	VEGFR2	VEGFR3	VEGF-D	BLANK	BLANK	BLANK	BLANK	BLANK	BLANK	BLANK	BLANK	POS

Supplementary Figure 3. Cytokine array

Relative 96 cytokine expression levels in the mouse serum (n = 6 per group) were analyzed using RayBio Mouse Cytokine Antibody Array Kits

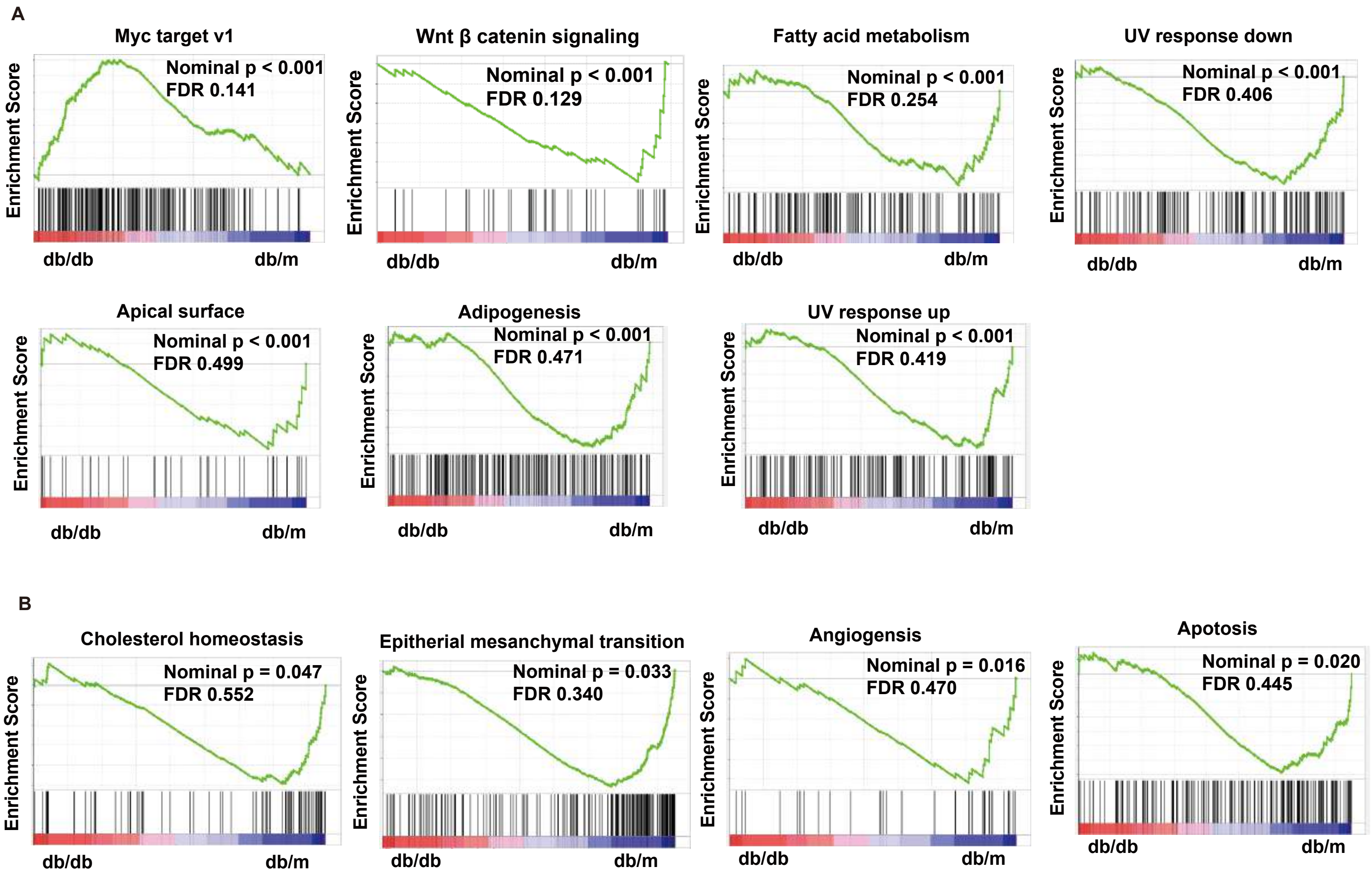

Supplementary Figure 4. GSEA analysis in skeletal muscle and small intestine.

The top of the figure plots the enrichment score for each gene, while the bottom of the plot shows the value of the ranking metric moving down the list of ranked genes in **(A)** skeletal muscle and **(B)** small intestine. y-axis: value of ranking metric; x-axis: rank of all genes.

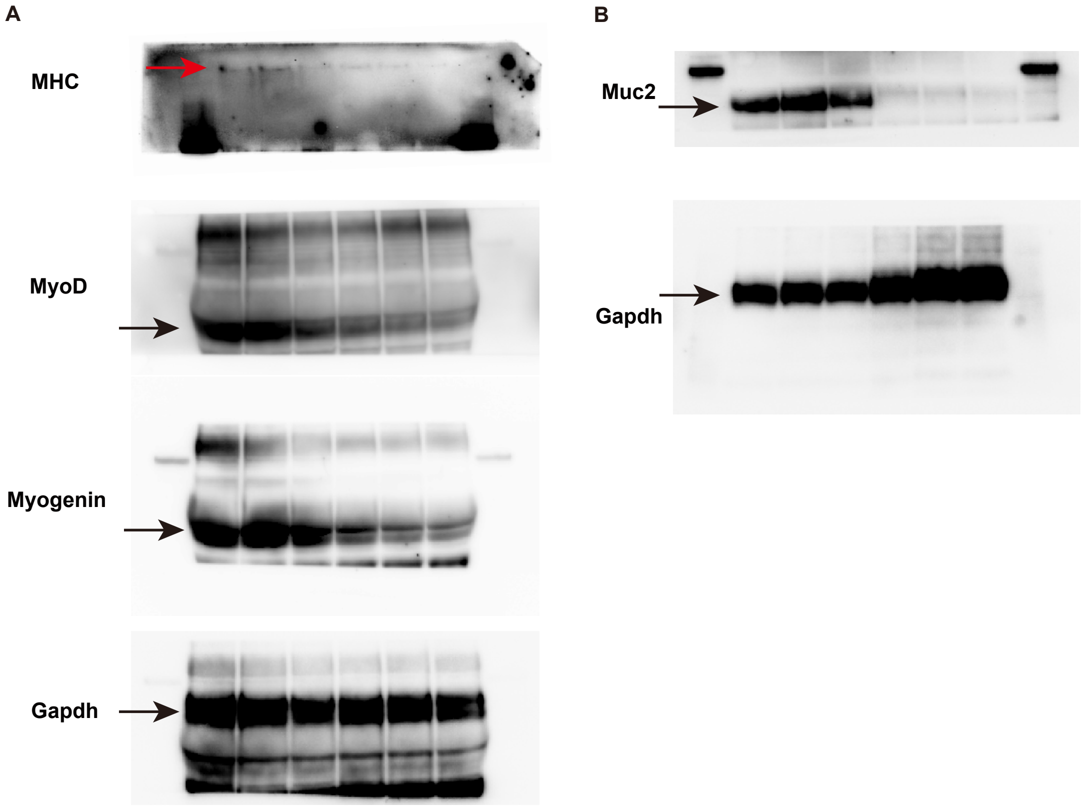

Supplementary Figure 5. Full gels for Western blot images of skeletal muscle and small intestine for quantification in Figure 2E and 3F.

Full gels of (A) skeletal muscle and (B) small intestine.

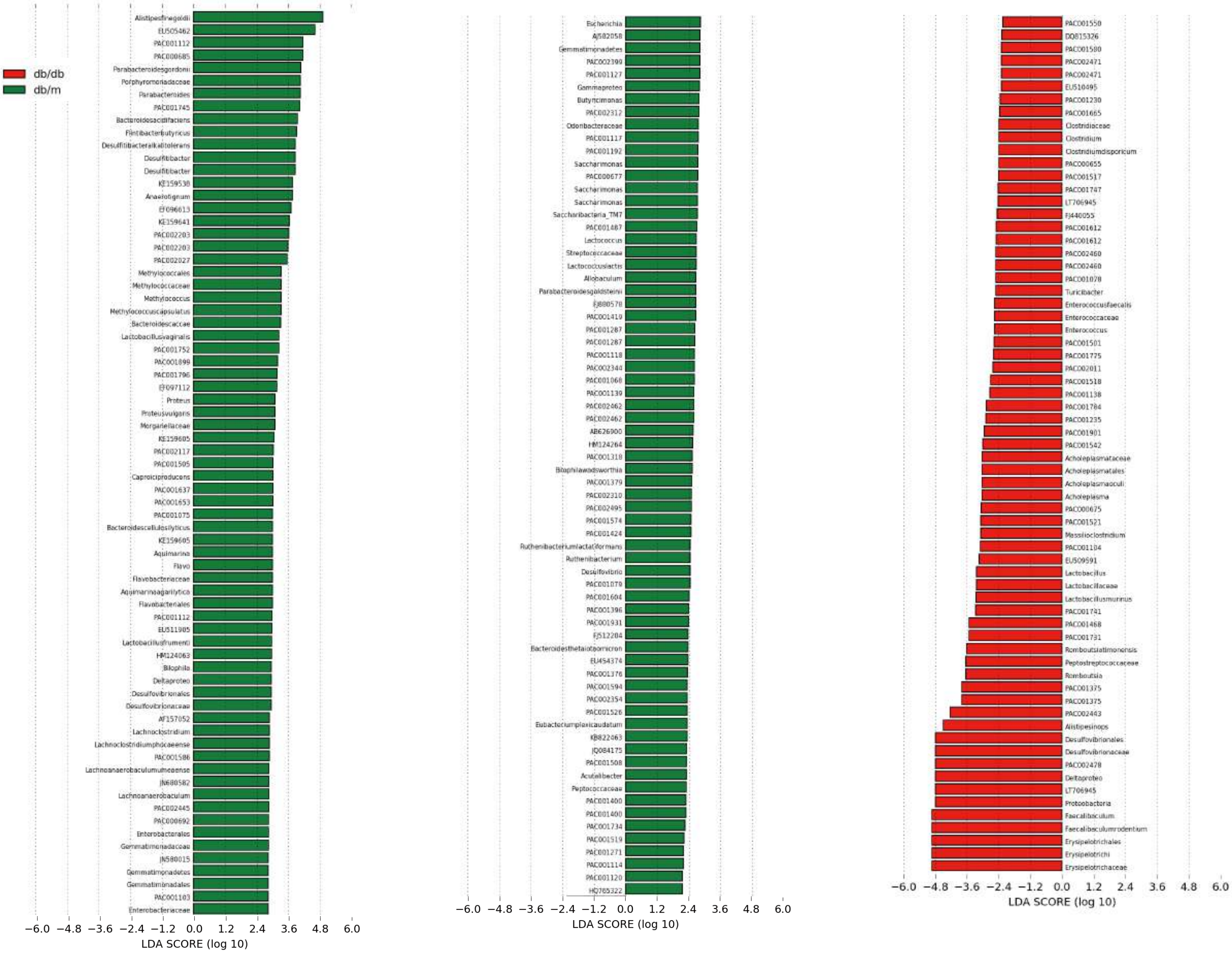

Supplementary Figure 6. LADA scroes

LDA scores of gut microbiota of db/m (green) and db/db (red) groups.

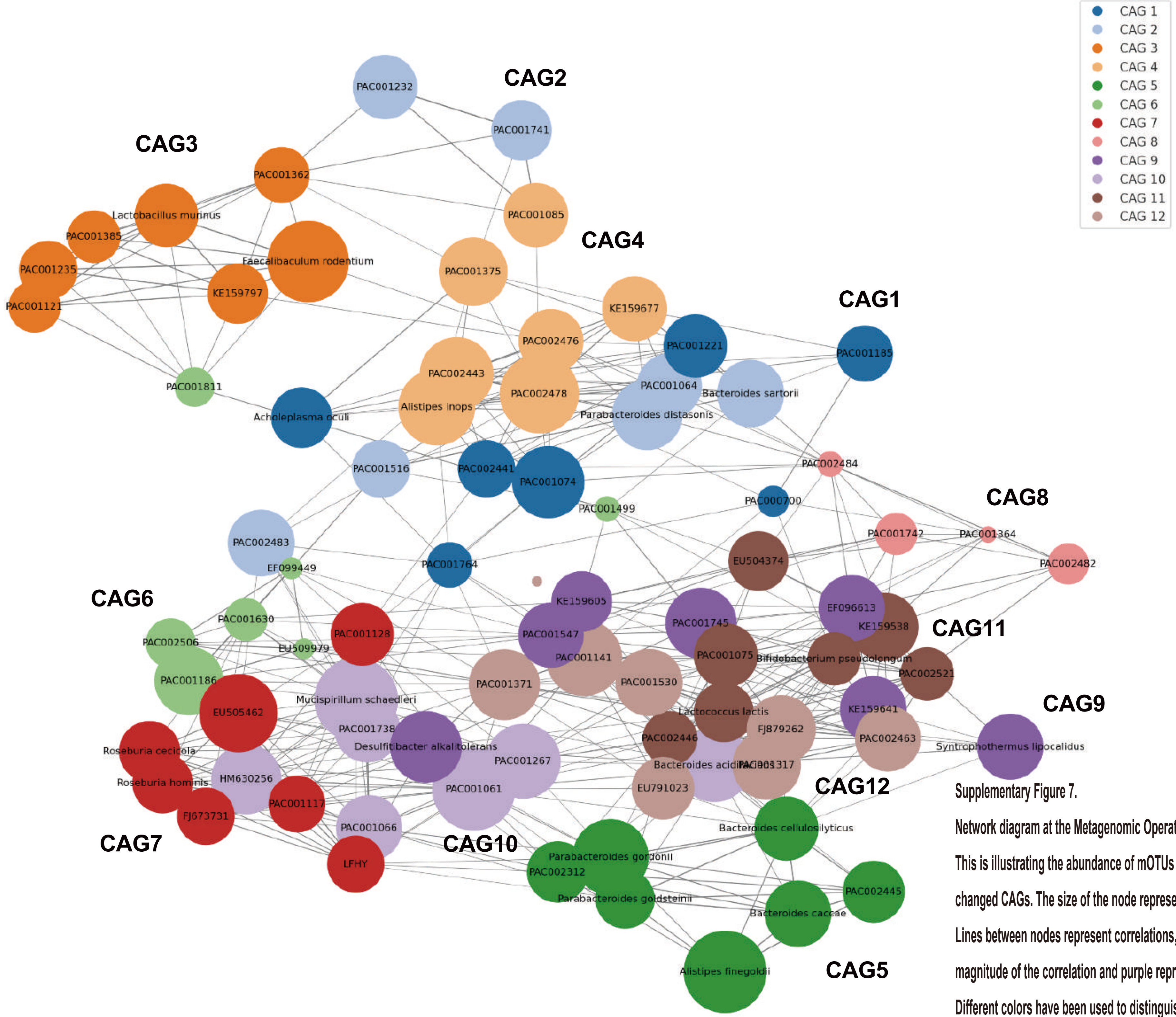

Supplementary Figure 7.

Network diagram at the Metagenomic Operational Taxonomic Unit (mOTU) level.

This is illustrating the abundance of mOTUs in different groups, based on significantly changed CAGs. The size of the node represents the average abundance of each mOTU. Lines between nodes represent correlations, with the width of the line indicating the magnitude of the correlation and purple representing a positive correlation.

Different colors have been used to distinguish CAGs.

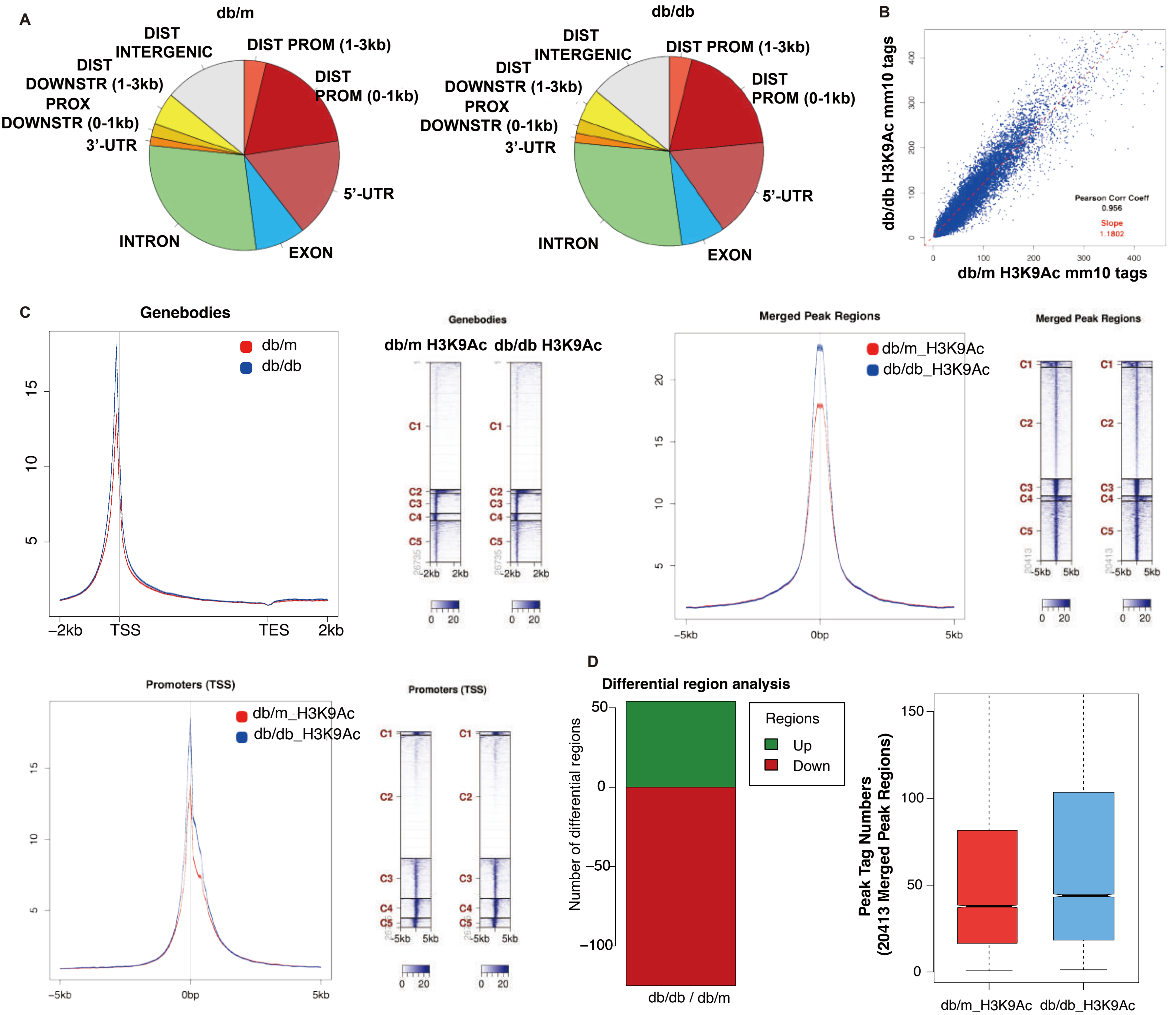

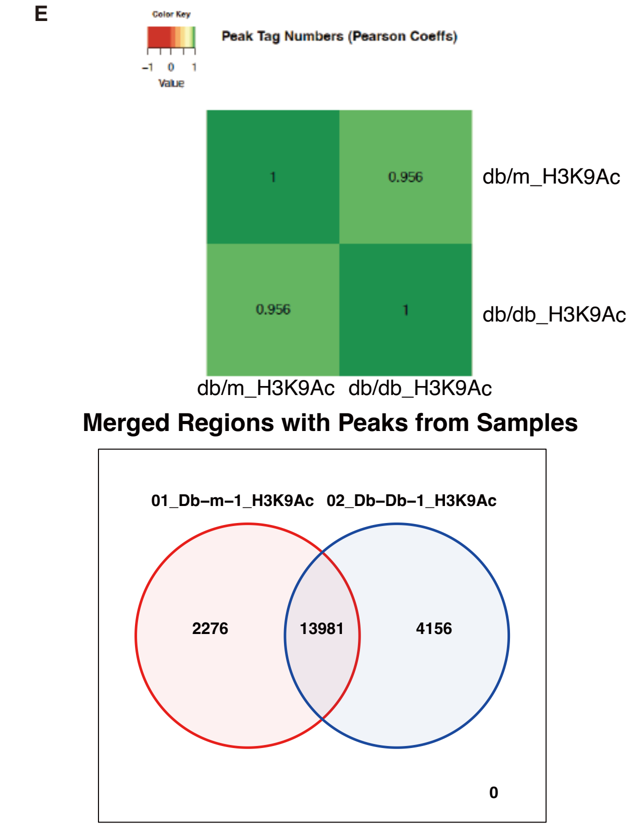

Supplementary figure 8. Global histone modifications via gene bodies in small intestinal epithelial cells. (A) Global occupancy of histone modification H3K9ac in different regions of the genome and the numbers were the number of peaks for each histone mark. (B) For each pairwise comparison, a scatter plot was generated plotting the tag numbers of db/m sample against db/db sample for each merged region. In addition, the slope is a measure for the average ratio of tag numbers between the 2 samples. (C) Tag distributions across target regions such as gene bodies (2 Kb upstream and downstream of the transcription start site (TSS) and transcription end site (TES), respectively) are presented both as average plots (average of values for all target regions in y-axis) and as heatmaps (values in z-axis as color, regions in y-axis). For the heatmaps, the data is clustered using the k-means algorithm (5 clusters; indicated by C1-C5) and sorted by decreasing average values inside each cluster. Red: db/m, Blue: db/db. Tag distributions across target regions such as Merged Regions (= all peak regions; +/- 5 kb) are presented both as average plots (average of values for all target regions in y-axis) and as heatmaps (values in z-axis as color, regions in y-axis). For the heatmaps, the data is clustered using the k-means algorithm (5 clusters; indicated by C1-C5) and sorted by decreasing average values inside each cluster. Tag distributions across target regions such as TSS (+/- 5 kb) are presented both as average plots (average of values for all target regions in y-axis) and as heatmaps (values in z-axis as color, regions in y-axis). For the heatmaps, the data is clustered using the k-means algorithm (5 clusters; indicated by C1-C5) and sorted by decreasing average values inside each cluster. (D) The number of differential regions were shown (db/db compared with db/m), Green: up, Red: down. This plot compared the distribution of peak tag numbers between the samples. The boxed area represents the center two quartiles (notched line = median), while the whiskers show the top and bottom quartiles without outliers. (E) This heatmap showed the Pearson Correlation coefficients of all pairwise comparisons, colored from dark green (high correlation) to yellow and red (low or no correlation). The overlap between peaks was displayed in the VENN diagram.
