# Supplementary material for: A multi‐omics approach to overeating and inactivity‐induced muscle atrophy in db/db mice
Source: J Cachexia Sarcopenia Muscle. 2024 Jul 13;15(5):2030–45. doi: 10.1002/jcsm.13550 (PMC11446703; doi:10.1002/jcsm.13550)
Supplement: Supplementary file 5 — Data S1. Supporting Information [file JCSM-15-2030-s006.docx]

**Supplementary references**

S1. Hazarika S, Dokun AO, Li Y, Popel AS, Kontos CD, Annex BH. Impaired angiogenesis after hindlimb ischemia in type 2 diabetes mellitus: differential regulation of vascular endothelial growth factor receptor 1 and soluble vascular endothelial growth factor receptor 1. *Circ Res* 2007;**101**:948–956.

S2. Byrne AM, Bouchier-Hayes DJ, Harmey JH. Angiogenic and cell survival functions of vascular endothelial growth factor (VEGF). *J Cell Mol Med* 2005;**9**:777–794.

S3. Celletti FL, Waugh JM, Amabile PG, Brendolan A, Hilfiker PR, Dake MD. Vascular endothelial growth factor enhances atherosclerotic plaque progression. *Nat Med* 2001;**7**:425–429.

S4. Zapata-Gonzalez F, Auguet T, Aragonès G, Guiu-Jurado E, Berlanga A, Martinez S *et al.* Interleukin-17A Gene Expression in Morbidly Obese Women. *Int J Mol Sci* 2015;**16**:17469–17481.

S5. Suárez-Álvarez K, Solís-Lozano L, Leon-Cabrera S, González-Chávez A, Gómez-Hernández G, Quiñones-Álvarez MS *et al.* Serum IL-12 is increased in Mexican obese subjects and associated with low-grade inflammation and obesity-related parameters. *Mediators Inflamm* 2013;**2013**:967067.

S6. Romanazzo S, Forte G, Morishima K, Taniguchi A. IL-12 involvement in myogenic differentiation of C2C12 in vitro. *Biomater Sci* 2015;**3**:469–479.

S7. Nunemaker CS, Chung HG, Verrilli GM, Corbin KL, Upadhye A, Sharma PR. Increased serum CXCL1 and CXCL5 are linked to obesity, hyperglycemia, and impaired islet function. *J Endocrinol* 2014;**222**:267–276.

S8. Atanes P, Lee V, Huang GC, Persaud SJ. The role of the CCL25-CCR9 axis in beta-cell function: potential for therapeutic intervention in type 2 diabetes. *Metabolism* 2020;**113**.

S9. Yuille S, Reichardt N, Panda S, Dunbar H, Mulder IE. Human gut bacteria as potent class I histone deacetylase inhibitors in vitro through production of butyric acid and valeric acid. *PLoS One* 2018;**13**.
