# Supplementary material for: A multi‐omics approach to overeating and inactivity‐induced muscle atrophy in db/db mice
Source: J Cachexia Sarcopenia Muscle. 2024 Jul 13;15(5):2030–45. doi: 10.1002/jcsm.13550 (PMC11446703; doi:10.1002/jcsm.13550)
Supplement: Supplementary file 7 — Data S3. Supporting Information [file JCSM-15-2030-s007.docx]

**Methods**

*Murine Models*

Male *db/m* and *db/db /m*urine models, aged seven weeks, were procured from Shimizu Laboratory Supplies, Kyoto, Japan, and sustained in a strictly controlled, pathogen-free environment. Littermate models, originated from Shimizu Laboratory Supplies, were incorporated within the experimental framework. The subjects, individually housed, were provisioned with a standard nutritional regimen (ND; constituting 345 kcal/100 g, with fat representing 4.6% of kcal; sourced from CLEA, Tokyo, Japan) for a duration of eight weeks, commencing at eight weeks of age, allowing unrestricted consumption. The sample size was computed utilizing EZR, employing relative grip strength as a referential metric. Given a mean discrepancy of 3.3 between the groupings, a standard deviation of 0.12, a predetermined significance threshold at 0.05, and a power determination at 80%, the necessitated sample size was identified as six. Consequently, the sample size was established at six.

*Quantification of Voluntary Wheel Running*

Murine subjects were singularly accommodated within enclosures, each equipped with a running wheel (Model MK-713; Muromachi Kikai). The cumulative rotations of the incorporated running wheel were meticulously logged throughout each nocturnal 12-hour phase, utilizing specialized software (CompACT AMS Ver.3: Muromachi Kikai) interfaced with the computational apparatus connected to the respective running wheels. The assessment of locomotor activity was diligently performed for a quintet of days preceding the scheduled euthanasia.

*Analytical Methodologies and Tolerance Tests for Glucose and Insulin*

Murine models, aged fifteen weeks, underwent intraperitoneal glucose tolerance evaluations (iPGTT) (administered at 2 g/kg of bodily mass) subsequent to a fasting period of 16 hours and were subjected to insulin tolerance assessments (ITT) (dispensed at 0.5 U/kg of body weight) following a 5-hour fast. Venous blood specimens were procured from the caudal vein. Glycemic indices were ascertained employing a glucometer (Model Gultest mint II; Sanwa Kagaku Kenkyusho, Nagoya, Japan). Distinct murine models were utilized for the execution of iPGTT and ITT. Glycemic indices were meticulously monitored at intervals of 0, 15, 30, 60, and 120 minutes post-injection. The area beneath the curve (AUC) of the yielded iPGTT and ITT data was subjected to analytical scrutiny.

*Assessment of grip strength*

The grip strength was quantified utilizing a specialized grip strength meter designated for murine models (Model DS2-50N, IMADA Co., Ltd, Toyohashi, Japan) in a distinct batch of murine models aged sixteen weeks. A series of six consecutive evaluations were conducted daily at intervals of one minute. The examiners were maintained in a state of blinding relative to the categorization of the murine models. The muscular grasp force was regularized to body mass.

*Serum Biochemical Analysis*

Blood specimens were procured from fasted murine models via cardiac perforation during the process of euthanasia, and the serum specimens were isolated subsequent to centrifugation conducted at 14,000 revolutions per minute for a duration of 10 minutes at 4 °C. The isolated serum was preserved at −30 °C pending dispatch to an analytical subcontractor. Alanine aminotransferase (ALT) concentrations were ascertained employing the standardized protocol delineated by the Japanese Society for Clinical Chemistry ^1^. Concentrations of triglycerides (TG) and total cholesterol were quantified utilizing enzymatic methodologies (TG, GK-GPO) ^2^. Biochemical evaluations (n = 6) were executed at the FUJIFILM Wako Pure Chemical Corporation (Osaka, Japan).

*Histopathological Examination of Hepatic and Epididymal White Adipose Tissues (eWAT)*

Liver tissues, meticulously extracted from murine models, were promptly conserved in 10% buffered formalin and 4% buffered paraformaldehyde for a duration of 24 hours at 22 °C, followed by embedding in paraffin. They were then sectioned into 4 µm thick slices and underwent both hematoxylin and eosin (HE) and oil red-O staining protocols. To assess the degree of nonalcoholic fatty liver disease (NAFLD) rigorously, the NAFLD activity score (NAS) was ascertained, serving as a distinguished criterion for appraising the intensity of nonalcoholic steatohepatitis (NASH) and modifications in NAFLD ^3^. The region exposed to oil red-O staining was quantitatively analyzed employing ImageJ software (Version 1.53k, NIH, Bethesda, MD, USA). The eWAT, upon meticulous excision from murine models, was instantaneously conserved in 10% buffered formalin and 4% buffered paraformaldehyde for a period of 24 hours at 22 °C, subsequently embedded in paraffin, partitioned into sections of 4 µm thickness, and underwent hematoxylin and eosin (HE) staining procedures. The adipocyte region was quantified through the utilization of ImageJ software (NIH, MD, USA).

*Cytokine Antibody Arrays*

The relative expression levels of 96 cytokines within mouse serum (n = 6) were meticulously assessed utilizing RayBio Mouse Cytokine Antibody Array Kits (Raybiotech, Norcross, GA, USA), strictly adhering to the manufacturer’s protocol. The array membrane signals were discerned by ImageQuant LAS 800 (GE Healthcare, Piscataway, NJ, USA), and the primitive data were replicated and integrated into the RayBio® Antibody Array Analysis Tool. Signals emanating from *db/m* mice were allocated a relative value of 1.0.

*Histopathological Examination of Soleus Muscular Tissue*

Tissue from the soleus muscle was procured from euthanized murine models, conserved in 10% buffered formaldehyde, and subsequently embedded within paraffin. Sections of muscular tissue were meticulously prepared and stained with hematoxylin and eosin (HE). The muscular tissues were sectioned at the point of maximal prominence. Imagery was obtained, and cross-sectional domains were quantified employing a BZ-X710 fluorescence microscope (Keyence, Osaka, Japan). The cross-sectional area was quantified through the utilization of ImageJ software (n= 6).

*Analysis of mRNA Sequencing of the Plantaris Muscular Tissue and Jejunum*

The plantaris muscle and jejunum, from murine models that underwent a 16-hour fasting period, were excised and instantaneously preserved in liquid nitrogen. The specimens were homogenized in ice-cold QIAzol Lysis Reagent (Qiagen, Venlo, The Netherlands) at 4000 revolutions per minute for a duration of 2 minutes within a ball mill, followed by the extraction of total RNA in accordance with the manufacturer’s prescribed protocol. A complementary DNA (cDNA) library was synthesized utilizing the TruSeq® Stranded mRNA kit (Qiagen, Carlsbad, CA, USA). Paired-end sequencing was executed on the Illumina NovaSeq6000 platform (n = 6). To scrutinize the functional enrichment of prolific genes within cells and nuclei, each gene was ranked based on log fold change, and gene set enrichment analyses (GSEA) were conducted on gene markers derived from prior studies ^4^. Within these analyses, a positive normalized enrichment score (NES) signifies gene enrichment, whereas a negative NES denotes gene depletion.

*Protein Extraction and Western Blot Analysis*

Derivatives of gastrocnemius muscle and small intestine were prepared in radioimmunoprecipitation assay buffer (RIPA; ATTO, Tokyo; containing 50 mmol/L Tris (pH 8.0), 150 mmol/L NaCl, 0.5% deoxycholate, 0.1% SDS, and 1.0% NP-40) amalgamated with a protease inhibitor cocktail (BioVision, Milpitas, CA, USA). The quantification of protein was executed utilizing a BSA protein assay kit (Pierce/Thermo Scientific), adhering to the manufacturer’s specified protocol. Total protein (40 mg) was subjected to electrophoresis in 15% SDS-PAGE gels for MyoD, Myogenin, and Gapdh and 5% SDS-PAGE gels for Myosin Heavy Chain and Muc2, followed by the implementation of Western blotting in accordance with standardized methodologies, and the visualization of protein was enabled by the ChemiDoc MP Imaging System (Bio-Rad Laboratories, Hercules, CA, USA). The quantification of signals was conducted via Image Lab software (Bio-Rad), with fold alterations ascertained as the ratio between the optical density of each protein normalized to Gapdh.

Protein isolation was carried out on the gastrocnemius muscle and small intestine. Initially, 40–60 µg of extracted protein was incubated with specific primary antibodies; namely, Myosin Heavy Chain (MAB4470, R&D Systems, Minneapolis, MN) (1:1000), MyoD (sc-377460, Santa Cruz Biotechnology, Santa Cruz, CA) (1:1000), and Myogenin (sc-12732, Santa Cruz Biotechnology) (1:1000) for gastrocnemius muscle, and Muc2 (27675-1-AP , proteintech, Rosemont, IL) (1:1000) for small intestine, or Gapdh (60004-1-Ig, proteintech) (1:1500) diluted with EzBlock Chemi (ATTO, Osaka, Japan) overnight at 4°C. This preceded incubation with secondary goat anti-mouse IgG antibodies conjugated to horseradish peroxidase, diluted with EzBlock Chemi for 60 minutes at room temperature.

*Histopathological Evaluation of Jejunum and Colon*

Jejunum and colon samples, meticulously excised from murine models, were promptly conserved in 10% buffered formaldehyde and Carnoy’s solution for a period of 24 hours at 22 °C. Following this, they were embedded in paraffin, partitioned into 4 µm-thick sections, and underwent staining procedures with hematoxylin and eosin (HE) and periodic acid Schiff (PAS) stain. Visualization of the stained sections was achieved using a fluorescence microscope (BZ-X710; Keyence). The proportion of villus height to width and the crypt depth were ascertained using HE-stained sections at five distinct locations per slide for each aggregation of 10 specimens, leveraging ImageJ software (Version 1.53 k, NIH, Bethesda, MD, USA). Mucin granules and goblet cells (PAS+) were quantified and represented as the average count of goblet cells (PAS+) per 10 crypts using ImageJ software, as formerly specified (n = 6) ^5^.

*Isolation of Mononuclear Cells from Murine Small Intestine and Flow Cytometric Analysis*

To avert blood contamination within the small intestine, a systemic perfusion with heparinized saline was performed preceding the extraction or lavation of the tissue with PBS. Samples were preserved in chilled 2% FBS in RPMI until experimental deployment. Subsequent investigations were executed on the day of euthanasia: Intestinal lamina propria (LPL) mononuclear cells were procured utilizing the Lamina Propria Dissociation Kit (130-097-410; Miltenyi Biotec, Germany), adhering to the manufacturer’s instructions. Cell conglomerates were resuspended in 5 mL of 40% Percoll®, and the cellular mixture was meticulously overlaid atop the centrifuge tubes containing a foundational layer of 5 mL of 80% Percoll®. Density gradient centrifugation (420×g, 20 min) was employed, and mononuclear cells located within the intermediate layer were carefully harvested utilizing a 1 mL pipette. The acquired mononuclear cells underwent dual rinses with 2% FBS/PBS.

*Quantification of Metabolites in Serum, Skeletal Muscle, and Feces*

Accumulated samples were preserved at -30 °C until required for experimental engagement. Serum (25 µL), obtained through cardiac puncture during euthanasia, fecal matter from the small intestine (15 µg), and skeletal muscle tissue samples (15 µg) were employed for the determination of free fatty acid concentrations. A fatty acid methylation kit (Nacalai Tesque, Kyoto, Japan) enabled the methylation analysis of the samples. Gas chromatography-mass spectrometry (GC-MS) analyses were conducted using an Agilent 7890B/7000D system (Agilent Technologies, Santa Clara, CA, USA) to assess levels of palmitic acid within murine serum, fecal matter, and skeletal muscle tissues (n= 6). The derived product was applied to a Varian capillary column (DB-FATWAX UI; Agilent Technologies). The capillary column allocated for fatty acid separation was CP-Sil 88 for FAME (100 m × 0.25 mm [internal diameter] × 0.20 µm [film thickness]; Agilent Technologies). The column was maintained at 100 °C for a period of 4 minutes, following which the temperature was progressively increased by 3 °C/min to 240 °C and sustained for an additional 7 minutes. Samples were injected in split mode with a specified split ratio of 5:1. Each fatty acid methyl ester was identified in the selected ion-monitoring mode. All the outcomes were normalized to the peak height for the C17:0 internal standard ^6^.

*Quantification of Amino Acid and Organic Acid Concentrations in Serum and Skeletal Muscle Samples, and Short-Chain Fatty Acid (SCFAs) Concentrations in Serum and Faecal Samples*

The compositional analysis of amino acids and organic acids in murine serum and gastrocnemius muscle, along with SCFAs in serum and faecal matter, was discerned utilizing gas chromatography-mass spectrometry (GC/MS) performed on an Agilent 7890B/7000D system (Agilent Technologies, Santa Clara, CA, USA).

We homogenized 20 mg of gastrocnemius muscle and feces in 500 µL of acetonitrile and 500 µL of distilled water by grinding in a ball mill (4000 rpm for 2 min). The serum samples were not ball-milled before proceeding to the next step. The samples were then shaken at 1000 rpm for 30 min at 37 °C and centrifuged at 14,000 rpm for 3 min at room temperature. The supernatant (500 μL) was separated, and 500 μL of acetonitrile was added to it; the mixture was shaken at 1000 rpm for 3 min at 37 °C. After centrifugation at 14,000 rpm for 3 min at room temperature, the pH of the mixture was adjusted to 8 with 0.1 mol/L NaOH, after which amino acids, organic acids, and SCFAs were extracted. The concentrations of amino acids, organic acids, and SCFAs were determined using GC/MS employing the online solid-phase extraction (SPE) method. In the SPE-GC system SGI-M100 (AiSTI SCIENCE, Wakayama, Japan), SPE and injection into the GC/MS system were automatically performed after the sample was added to the vial and set on an autosampler tray. Flash-SPE ACXs (AiSTI SCIENCE) was used for solid phase stratification. To measure the concentrations of amino acids and organic acid, 50 µL aliquots of each of the sample extracts were loaded onto the solid phase and washed with acetonitrile and water (1:1). The samples were then dehydrated with acetonitrile and impregnated with 4 μL of 0.5% methoxyamine–pyridine solution. Thereafter, N-methyl-N-trimethylsilyltrifluoroacetamide was supplied to the solid phase to perform methoxylation and trimethylsilylation during derivatization and eluted with hexane. The final product was injected through the PTV injector, LVI-S250 (AiSTI SCIENCE), and the temperature was maintained at 220 °C for 0.5 min, increased gradually at 50 °C/min to 290 °C, and then held there for 16 min. The samples were loaded onto a capillary column, Vf-5 ms (30 m × 0.25 mm [inner diameter] × 0.25 μm [membrane thickness]; Agilent Technologies). The column temperature was maintained at 80 °C for 3 min, then increased gradually at 25 °C/min to 190 °C, at 3 °C/min to 220 °C, and at 15 °C/min to 310 °C, and held there for 4.6 min. The sample was injected in the split mode at a split ratio of 50:1. The samples were then dehydrated with acetone, impregnated with 4 μL of N-tert-butyldimethylsilyl-N-methyltrifluoroacetamide–toluene solution (1:3), and eluted with hexane after derivatization on the solid phase. The final product was injected through the PTV injector, LVI-S250, and the temperature was maintained at 150 °C for 0.5 min, increased gradually at 25 °C/min to 290 °C, and then held there for 16 min. The samples were loaded onto a capillary column, Vf-5 ms (30 m × 0.25 mm [inner diameter] × 0.25 μm [membrane thickness]; Agilent Technologies). The column temperature was maintained at 60 °C for 3 min, increased gradually at 10 °C/min to 100 °C and at 20 °C/min to 310 °C, and then held there for 7 min. The sample was injected in the split mode at a split ratio of 20:1. Amino acids, organic acids, and SCFAs were detected in the scan mode (m/z, 70–470). All results were normalized to the peak heights of norleucine (0.01 mM) for amino acids and organic acids ^7^ and to those of tetradeuteroacetic acid for SCFAs (0.02 mM) ^8^.

*Analysis of Gut Microbiota Composition*

Fecal samples were meticulously obtained from the appendix and subsequently allocated to cryotubes. Immediately after procurement, the samples underwent cryopreservation in liquid nitrogen and were maintained in this state until the DNA extraction process. From the appendix of three distinct mice, three fecal samples were individually collected, intentionally excluding one small and one large mouse housed within each group’s enclosure. Microbial DNA was assiduously isolated from the cryopreserved fecal samples using the QIAamp® DNA Stool Mini Kit (Qiagen, Venlo, Netherlands), adhering rigorously to the manufacturer’s specified protocol.

Whole-genome shotgun sequencing was performed using a HiSeq 2000/2500/4000 system (Illumina) at Bioengineering Lab. Co., Ltd., Sagamihara, Japan). To refine sequence quality, QIIME version 1.9.1 was applied. Barcodes or primers exhibiting scores below 75% were meticulously removed from the files. The identification of operational taxonomic units (OTUs) was conducted using the UCLUST algorithm, with a 97% similarity threshold ^9^. In addition, to assess whether the sampling effort was adequate to detect the majority of the gut bacterial community in each group, sequence coverage was calculated using Good's coverage ^10^. Predictions of Kyoto Encyclopedia of Genes and Genomes (KEGG) ortholog abundance were determined using the Phylogenetic Investigation of Communities by Reconstruction of Unobserved States (PICRUSt2) software ^11^. The relative abundance of phyla within the cohorts was evaluated using one-way ANOVA, accompanied by a Holm–Šídák multiple-comparison test. Alpha diversity, representing the diversity within a single sample, was assessed using the Chao1 ^12^, Shannon ^13^, and Gini–Simpson^14^.

The comparative abundance of bacterial genera between groups was analyzed using Linear Discriminant Analysis (LDA) combined with effect size measurements (LEfSe) (<http://huttenhower.sph.harvard.edu/lefse/>, accessed on May 15, 2023) ^15^. LEfSe, processing a normalized relative abundance matrix, identified taxa exhibiting significant abundance differences, and the effect size of each feature was assessed through LDA. A p-value threshold of 0.05 (Wilcoxon rank-sum test) along with an effect size threshold of 2 were established for all biomarkers identified in this study.

*Network Analysis and Visualization Methodology*

Co-abundance gene groups (CAGs) were identified to elucidate the potential groups of bacteria that tend to be abundant or scarce concurrently within the microbial communities. The network analysis and visualization were conducted using Python, utilizing Pandas for data manipulation, NetworkX for network analysis, and Matplotlib for visualization. Initially, the correlation data were filtered to retain only pairs with a correlation coefficient ≥ 0.4. Subsequently, within each CAG, bacteria were ranked based on mean abundance, and the top 7 were selected for inclusion in the network visualization. A graph was then constructed where nodes represented the selected bacteria and edges represented significant correlations between them, with edge width proportional to the correlation coefficient. Node sizes were determined by the log2-transformed Mean Abundance values of the corresponding bacteria, scaled for visual clarity, and were colored distinctly according to their CAG, using a predefined color palette (matplotlib’s tab20). The graph employed the Kamada-Kawai layout algorithm for node positioning, optimizing the distance between all pairs of nodes to create a balanced and readable visualization. The final visualization included a legend indicating the color corresponding to each CAG, and various custom adjustments were applied to node sizes, edge widths, and colors to enhance clarity and aesthetics.

*NON-TiE-UP Cleavage Under Targets and Tagmentation*

Small intestinal epithelial cells were isolated using previously published protocols ^16^. The foundational kit used for CUT&Tag was a CUT&Tag-IT Assay Kit (Active Motif). After rinsing the zona-free blastocysts with phosphate-buffered saline combined with 0.01% (w/v) polyvinyl alcohol and 1% (v/v) Protease Inhibitor Cocktail (PIC), they were individually placed into Antibody Buffer, enriched with the target primary antibody, digitonin, and PIC, situated in a round bottom-shaped 96-well plate. An overnight incubation at 4 °C with gentle agitation was employed, with a negative control being established by omitting the primary antibodies. Following primary antibody interaction, blastocysts were moved to wells containing Dig-Wash buffer, a secondary antibody, digitonin, and PIC, and were incubated at room temperature. After washes, the blastocysts were relocated to wells containing Dig-300 Buffer, pA-Tn5 transposomes, digitonin, and PIC, and once again incubated at room temperature. After washes, blastocysts were moved to individual microcentrifuge tubes containing Tagmentation Buffer with digitonin and PIC and incubated at 37 °C. After tagmentation, EDTA, SDS, and proteinase K were added and underwent a series of incubation and heating steps. SPRIselect beads were incorporated, vortexed, and incubated, followed by magnetic separation and subsequent washes with ethanol. After drying, DNA Purification Elution Buffer was added, and the liquid containing tagmented DNA was collected. PCR amplification of sequencing libraries was conducted using tagmented DNA and indexing primers according to the manufacturer’s instructions. The PCR libraries experienced a series of temperature conditions, followed by post-PCR library purification with SPRIselect beads and ethanol washes. The sequencing libraries were ultimately eluted in DNA Purification Elution Buffer. The paired-end 38 bp sequence reads (PE38) obtained by Illumina Sequencing were aligned to the genome using the default settings of the BWA algorithm, and alignment information was stored in BAM format. Only reads satisfying specific criteria were considered for subsequent analyses, and duplicate reads were removed. The MACS3 peak calling algorithm identified genomic regions with high transposition/tagging events, and fragment density was assessed by dividing the genome into specified bins and determining the number of fragments within ^17^.

**Referecnes**

1. Kotani K, Maekawa M KT. [Reestimation of aspartate aminotransferase (AST)/alanine aminotransferase (ALT) ratio based on JSCC consensus method--changes of criteria for a differential diagnosis of hepatic disorders following the alteration from Karmen method to JSCC method] - PubM. *Nihon Shokakibyo Gakkai Zasshi* 1994;**91**:154–161.

2. McGowan MW, Artiss JD, Strandbergh DR ZB. A peroxidase-coupled method for the colorimetric determination of serum triglycerides - PubMed. *Clin Chem* 1983;**29**:538–542.

3. Kleiner DE, Brunt EM, van Natta M, Behling C, Contos MJ, Cummings OW *et al.* Design and validation of a histological scoring system for nonalcoholic fatty liver disease. *Hepatology* 2005;**41**:1313–1321.

4. Subramanian A, Tamayo P, Mootha VK, Mukherjee S, Ebert BL, Gillette MA *et al.* Gene set enrichment analysis: a knowledge-based approach for interpreting genome-wide expression profiles. *Proc Natl Acad Sci U S A* 2005;**102**:15545–15550.

5. Motta JP, Flannigan KL, Agbor TA, Beatty JK, Blackler RW, Workentine ML *et al.* Hydrogen sulfide protects from colitis and restores intestinal microbiota biofilm and mucus production. *Inflamm Bowel Dis* 2015;**21**:1006–1017.

6. Okamura T, Nakajima H, Hashimoto Y, Majima S, Senmaru T, Ushigome E *et al.* Low circulating dihomo-gamma-linolenic acid is associated with diabetic retinopathy: A cross sectional study of kamogawa-dm cohort study. *Endocr J* 2021;**68**:421–428.

7. Nakajima H, Nakanishi N, Miyoshi T, Okamura T, Hashimoto Y, Senmaru T *et al.* Inulin reduces visceral adipose tissue mass and improves glucose tolerance through altering gut metabolites. *Nutr Metab (Lond)* 2022;**19**.

8. Kawano R, Okamura T, Hashimoto Y, Majima S, Senmaru T, Ushigome E *et al.* Erythritol ameliorates small intestinal inflammation induced by high-fat diets and improves glucose tolerance. *Int J Mol Sci* 2021;**22**.

9. Edgar RC. Search and clustering orders of magnitude faster than BLAST. *Bioinformatics* 2010;**26**:2460–2461.

10. Esty WW. The Efficiency of Good’s Nonparametric Coverage Estimator. *https://doi.org/101214/aos/1176350066* 1986;**14**:1257–1260.

11. Douglas GM, Maffei VJ, Zaneveld JR, Yurgel SN, Brown JR, Taylor CM *et al.* PICRUSt2 for prediction of metagenome functions. *Nat Biotechnol* 2020;**38**:685–688.

12. Chao A, Chazdon RL, Colwell RK, Shen TJ. Abundance-based similarity indices and their estimation when there are unseen species in samples. *Biometrics* 2006;**62**:361–371.

13. Shannon C.E. WW. The mathematical theory of communication. *University of Illinois Press* 1949;pp 1-117.

14. Simpson EH. Measurement of Diversity. *Nature 1949 163:4148* 1949;**163**:688–688.

15. N S, J I, L W, D G, L M, WS G *et al.* Metagenomic biomarker discovery and explanation. *Genome Biol* 2011;**12**.

16. Pan D, Das A, Liu D, Veazey RS, Pahar B. Isolation and characterization of intestinal epithelial cells from normal and SIV-infected rhesus macaques. *PLoS One* 2012;**7**.

17. Zhang Y, Liu T, Meyer CA, Eeckhoute J, Johnson DS, Bernstein BE *et al.* Model-based analysis of ChIP-Seq (MACS). *Genome Biol* 2008;**9**.
